# Auxin mediates the touch-induced mechanical stimulation of adventitious root formation under windy conditions in *Brachypodium distachyon*

**DOI:** 10.1186/s12870-020-02544-8

**Published:** 2020-07-16

**Authors:** Bo Eun Nam, Young-Joon Park, Kyung-Eun Gil, Ju-Heon Kim, Jae Geun Kim, Chung-Mo Park

**Affiliations:** 1 Department of Chemistry, Seoul National University, Seoul, 08826 South Korea; 2 Department of Biology Education, Seoul National University, Seoul, 08826 South Korea; 3 Plant Genomics and Breeding Institute, Seoul National University, Seoul, 08826 South Korea

**Keywords:** Adventitious root, Auxin, *Brachypodium distachyon*, Gravity, Lodging, Thigmomorphogenesis, Wind

## Abstract

**Background:**

It is widely perceived that mechanical or thigmomorphogenic stimuli, such as rubbing and bending by passing animals, wind, raindrop, and flooding, broadly influence plant growth and developmental patterning. In particular, wind-driven mechanical stimulation is known to induce the incidence of radial expansion and shorter and stockier statue. Wind stimulation also affects the adaptive propagation of the root system in various plant species. However, it is unknown how plants sense and transmit the wind-derived mechanical signals to launch appropriate responses, leading to the wind-adaptive root growth.

**Results:**

Here, we found that *Brachypodium distachyon*, a model grass widely used for studies on bioenergy crops and cereals, efficiently adapts to wind-mediated lodging stress by forming adventitious roots (ARs) from nonroot tissues. Experimental dissection of wind stimuli revealed that not bending of the mesocotyls but physical contact of the leaf nodes with soil particles triggers the transcriptional induction of a group of potential auxin-responsive genes encoding WUSCHEL RELATED HOMEOBOX and LATERAL ORGAN BOUNDARIES DOMAIN transcription factors, which are likely to be involved in the induction of AR formation.

**Conclusions:**

Our findings would contribute to further understanding molecular mechanisms governing the initiation and development of ARs, which will be applicable to crop agriculture in extreme wind climates.

## Background

Plants are constantly challenged with changes in surrounding environments throughout their life cycles. It has long been perceived by agricultural breeders and plant scientists that mechanical stimuli, such as physical touch by passing animals, insect attack, raindrop, wind, and flooding, profoundly affect plant growth and morphogenesis. This thigmomorphogenic process is considered to be an adaptation strategy that facilitates plants to cope with mechanical disturbances and thus is important for agricultural practice [1]. It is known that plant growth hormones, including auxin and ethylene, mediate the thigmomorphogenic responses [2–4].

Wind-induced mechanical stress is a representative thigmo stimulation in plants. Under changing natural conditions, wind often causes lodging of the stems and roots and disorientation of the leaves, leading to a significant yield loss of cereal crops [5]. Plants have evolved versatile adaptive strategies to manage these mechanical wind forces. The wind-responsive morphological changes allow plants to avoid stem breakage and uprooting by increasing plant mechanical strength and minimizing the disturbing force from wind [6].

A frequent consequence of wind-mediated mechanical disturbances is plant lodging, the dislocation of stems or roots from their proper, vertical placement. A number of studies has explored plant morphogenic traits that underlie lodging tolerance [7–9]. While elongated stems make plants more susceptible to lodging stress in rice [10], shorter stems are known to contribute to higher grain yields in barley [8]. Stem diameter and cell wall thickness also greatly affect lodging tolerance. It has been shown that stem thickness is positively correlated with lodging tolerance [11]. On the other hand, an increase of stem diameter reduces lodging tolerance in barley and oat [12]. There are many additional morphogenic traits that play roles in inducing lodging tolerance in cereals. Notably, the modes of plant lodging responses vary depending on the intensity and timing of lodging stimuli in different plant species [13, 14].

The morphological modifications under lodging stress are intimately associated with changes in biochemical characteristics, such as contents and depositions of lignin, cellulose, and sugar. An increase in cellulose and lignin contents improves stem wall thickness and flexibility. Accordingly, genes involved in lignin biosynthesis are induced during lodging process [15]. It is also known that higher starch contents help plants restore normal growth against wind-induced lodging stimulation [7].

Wind-driven plant responses to lodging stimulation also include the rearrangement of biomass to the root systems and the restructuring of the root growth patterning. It is known that the root/shoot ratio is increased, reinforcing the root anchorage system, during plant adaptation to wind stimulation [16]. Overall, it is evident that the aboveground wind-mediated mechanical forces profoundly affect the root growth and architecture. However, the wind-driven thigmomorphogenic responses of the root system have not been explored at the molecular level, mainly because the root system is influenced broadly by an extensive network of both the aboveground and soil conditions [17].

Recent studies have provided an invaluable hint for elucidating molecular mechanisms governing root thigmomorphogenesis. Adventitious roots (ARs) are formed from nonroot tissues not only as part of normal development but also as an adaptive strategy in response to environmental stresses [18]. In cereal crops, the ARs are important for plant adaptation to adverse stress conditions, such as flooding, drought, and soil burial. In recent years, there have been great advances in understanding molecular mechanisms underlying the formation of ARs in *Arabidopsis*. It has been suggested that AR formation is initiated not only from pericycle cells but also from various cell types [19]. In addition, it is known that a variety of plant growth hormones coordinates various steps of AR formation in conjunction with developmental and external stimuli in different model species [19]. While auxin is a master player in the AR formation, it also interacts with other growth hormones, such as ethylene, brassinosteroids, gibberellic acids (GA), abscisic acid (ABA), cytokinins, and jasmonic acid (JA), through a hub of signaling crosstalks [19].

In rice, AR development is initiated both during normal development and under stressful stimuli [20]. It is known that AR formation is mediated by LATERAL ORGAN BOUDARIES DOMAIN (LBD), ADVENTITIOUS ROOTLESS1 (ARL1), and WUSCHE RELATED HOMEOBOX11 (WOX11) transcription factors [21, 22]. Accordingly, ARL1-deficient rice mutants are incapable of forming ARs [21]. The *ARL1* gene is responsive to auxin and ethylene signals and expressed primarily in lateral roots and AR primordia, tiller primordia, and vascular tissues. Meanwhile, WOX11 plays a role in cytokinin and auxin signaling pathways during AR development [22]. It is involved in AR emergence and growth, coordinating auxin and cytokinin signaling cascades that stimulate cell division. However, it is currently unclear whether the auxin-mediated AR formation is linked with wind-induced thigmomorphogenic root responses.

In this study, we systematically investigated how *Brachypodium distachyon*, a widely used monocot model in recent years, adapts to wind-mediated lodging stress. It was found that in response to constant wind flow*, Brachypodium* develops shoot-born ARs from the leaf nodes, which strengthen the statue of the plant against stem and root lodging stress. Interestingly, direct contact of the leaf nodes with soil particles, not the bending of the mesocotyls itself, is the major stimulating cue that induces AR formation through WOX- and LBD-mediated auxin signaling pathways. Our findings demonstrate that the wind-induced stimulation of thigmomorphogenic AR formation is essential for the enhancement of lodging tolerance and rapid recovery from stem flattening in *Brachypodium* and perhaps related grass species as well.

## Results

### *Brachypodium* adapts to wind-induced lodging stress by forming ARs

On the basis of the previous observations that wind triggers thigmomorphogenic root responses and auxin plays a major role in inducing AR formation, we hypothesized that auxin-mediated AR formation is associated with wind-induced thigmomorphogenic adaptation in *Brachypodium*.

**Fig. 1:**
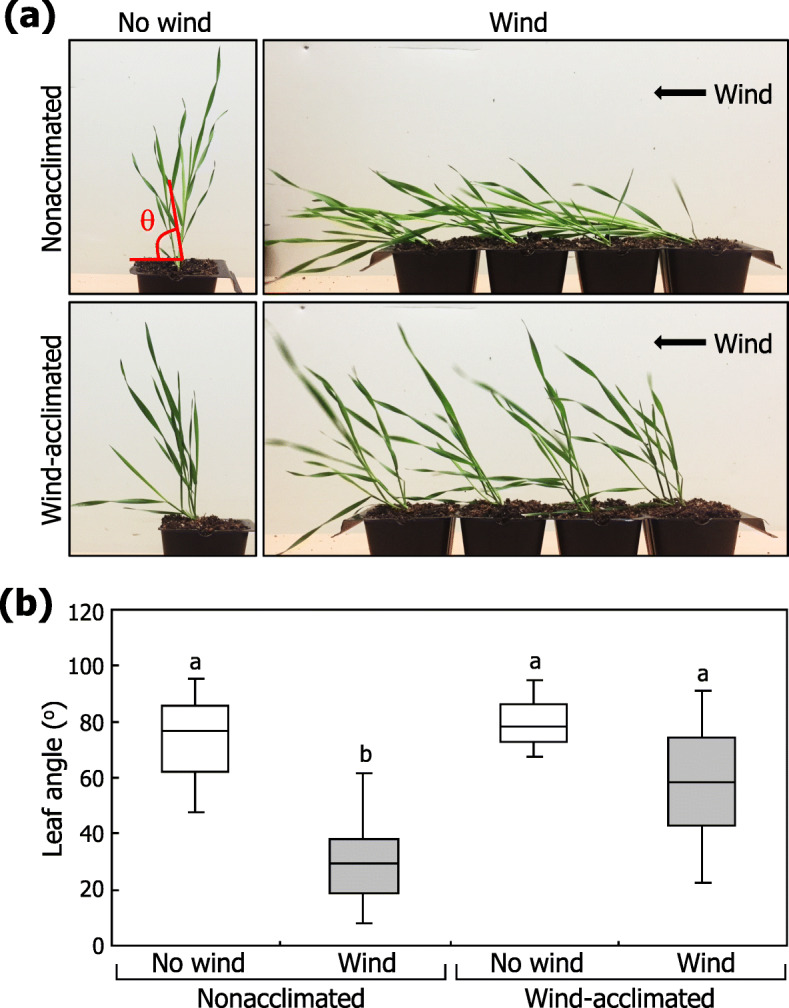
Adaptation of *Brachypodium* plants to wind-induced mechanical stimulation. Three-week-old plants grown in soil were either exposed to a constant unidirectional wind flow or grown under control conditions (no wind) for 10 days and then subjected to wind treatments (wind-acclimated or nonacclimated, respectively). **a** Lodging phenotypes in response to wind stimulation were analyzed by measuring the angles of lodged tillers relative to the soil surface (θ). **b** Wind-treated plants were photographed, and the largest angles were measured. Box plots show the range of the angles of lodged tillers (*n* > 20). Different letters represent a significant difference (*P* < 0.01) determined by the Tukey’s honestly significant difference (HSD) test

We first examined plant responses to wind-mediated mechanical stimulation. While nonacclimated plants exhibited stem lodging in response to wind flow, wind-acclimated plants exhibited a relatively reduced bending of the shoots (Fig. 1a; Additional file 1: Fig. S1). Quantitative examination of wind resistance by measuring the tiller angles relative to the horizontal plane revealed that while the tiller angle was approximately 30 degrees in nonacclimated plants, it was larger than 60 degrees in wind-acclimated plants (Fig. 1b). These observations indicate that *Brachypodium* is capable of adapting to wind-mediated lodging stress.

**Fig. 2:**
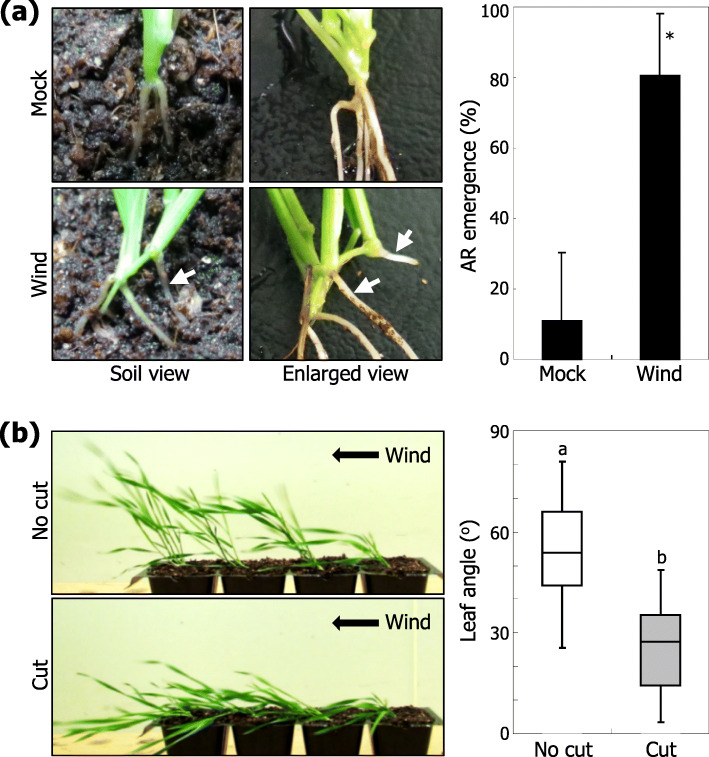
Induction of AR formation by wind stimulation. Three-week-old plants were either exposed to a unidirectional wind flow or grown under mock conditions (no wind) for 10 days prior to analyzing AR emergence. **a** AR emergence. ARs formed in the soil-grown plants and their enlarged views were displayed (left photographs). White arrows indicate ARs. Leaf node roots formed on the tillers were counted as ARs. Three independent experiments, each consisting of 16 plants, were statistically analyzed (*t*-test, *P < 0.01) (right graph). Error bars indicate standard error of the mean (SE). **b** Wind response of plants with or without ARs. Visible ARs of the unidirectional wind-treated plants were either retained (no cut) or cut out, and the plants were exposed to wind stimulation (left photographs). The angles of lodged tillers were statistically analyzed (right graph, *n =* 20). Different letters represent a significant difference (*P* < 0.01) determined by the Tukey’s HSD test

We next investigated growth and morphological changes that accompanied wind acclimation. Overall plant morphology and growth patterns in the aerial plant parts were not discernibly affected by wind treatments in our assay conditions (Fig. 1). Interestingly, we found that more ARs were formed from the leaf nodes of the tillers in wind-treated plants (Fig. 2a), raising a possibility that AR formation is responsible for the lodging-tolerant phenotype. To examine this possibility, we artificially removed the ARs of wind-acclimated plants and performed an additional round of wind treatments. While AR-retaining plants exhibited lodging tolerance, AR-removed plants exhibited a significant reduction of lodging tolerance (Fig. 2b). Together, these observations strongly support the notion that the wind-induced formation of ARs is functionally associated with lodging tolerance.

### Wind-driven falling down of the shoots triggers AR formation

A visible effect of wind on plants is the physical falling down of the aerial plant parts. Meanwhile, it is possible that wind causes changes in air humidity and plant body temperature, which would indirectly influence stem lodging. We first tested whether air humidity is affected by wind treatments. It was found that wind decreased the relative humidity of air by approximately 10% under our assay conditions (Additional file 2: Fig. S2a). On the other hand, the water status of the soil was not discernibly altered under the identical assay conditions (Additional file 2: Fig. S2b). The water content of plant leaves was also unaltered by wind treatments (Additional file 2: Fig. S2c). Furthermore, temperatures of both air and plant bodies were not markedly affected by wind treatments (Additional file 3: Fig. S3a, b). These observations indicate that our assay system is suitable for analyzing the effects of wind on the induction of AR formation.

**Fig. 3:**
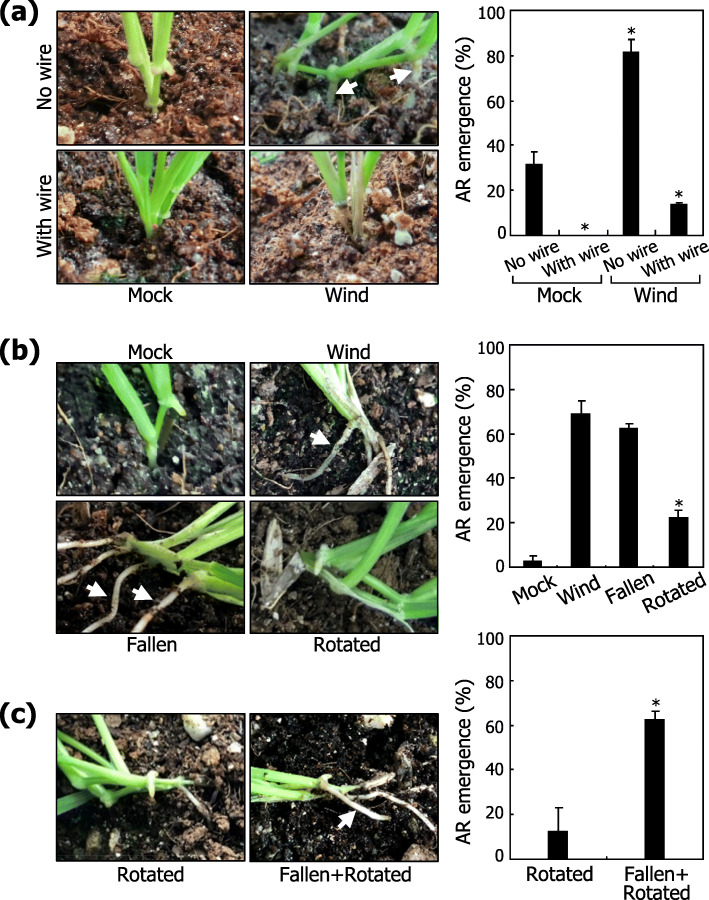
Effects of wind-driven falling down on AR formation. Three-week-old plants grown in soil were further grown for 10 days under various experimental conditions. Three independent experiments, each consisting of 16 plants, were statistically analyzed (*t*-test, *P < 0.01). Error bars indicate SE. White arrows indicate ARs. **a** Induction of AR formation by wind-induced lodging. Plants were exposed to wind flow with or without supporting wires, which protect plants from falling down. Following wind treatments, representative plants were photographed (left photographs), and AR emergence was statistically analyzed (right graph). **b** Effects of mechanical and gravity stimuli. Plants were artificially fallen down using wires (fallen). Plants were also grown on a slope of 75^o^ to impose gravity stimulation (rotated). Following treatments, representative plants were photographed (left photographs), and AR emergence was statistically analyzed (right graph). **c** Effects of a combined stimulation of falling down and gravity. Plants were artificially fallen down and then rotated by 75^o^ to impose a combined stimulation. Representative plants were photographed (left photographs), and AR emergence was statistically analyzed (right graph)

Wind is a complex environmental factor, which can be dissected into several simpler components (Additional file 4: Fig. S4). A critical question was which component of wind is the major determinant of AR formation against wind stimulation. Prior to wind treatments, *Brachypodium* plants were equipped with supporting wires so that the shoots do not fall down even under wind conditions (Additional file 5: Fig. S5a). Notably, plants equipped with supporting wires exhibited a significantly reduced emergence of ARs (Fig. 3a), indicating that wind-induced mechanical falling down of the shoots is functionally associated with the induction of AR emergence.

Physical bending of the mesocotyls, which are relatively flexible (Additional file 5: Fig. S5b), positions the leaf nodes horizontally, rotating the direction of gravity 90 degrees in these plant organs. Gravi-stimulation is known to induce lateral root emergence in *Arabidopsis* [23]. To examine the effects of gravity on AR formation, plants grown in soil were either artificially fallen down using arresting wires or rotated horizontally and further grown for 10 days (Additional file 5: Fig. S5c, d, respectively). Artificially fallen plants exhibited a high frequency of AR emergence similar to what observed in wind-treated plants (Fig. 3b). In contrast, rotated plants exhibited a markedly reduced frequency of AR emergence compared to those in wind-treated and artificially fallen plants. A combined stimulation of falling down and rotating treatments recovered the frequency of AR emergence comparable to that observed in wind-treated plants (Fig. 3c; Additional file 5: Fig. S5e). Together, these observations indicate that falling down of the shoots, not gravi-stimulation, play a major role in the induction of AR formation.

It was notable that in plants stimulated by artificial falling down and gravity, ARs were formed mostly from the soil-contacting side of the leaf nodes (Fig. 3c), suggesting that not the physical bending of the mesocotyls but the direct contact of the leaf nodes with soil particles is a primary determinant of the AR formation.

### Direct contact of the leaf nodes with soil particles triggers the induction of AR formation

**Fig. 4:**
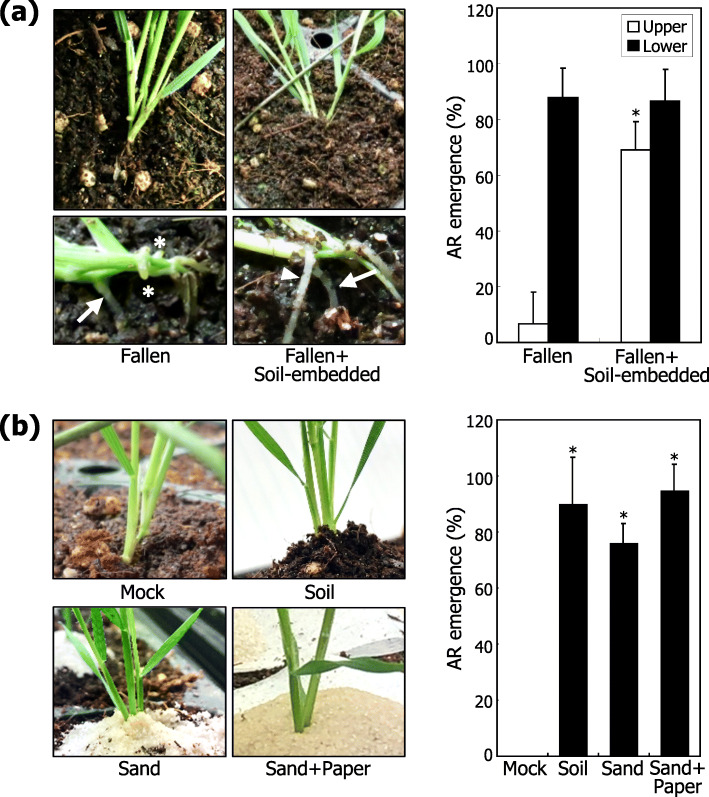
Induction of AR formation by physical soil contact. Three independent experiments, each consisting of 16 plants, were statistically analyzed using Student *t*-test (*P < 0.01). Error bars indicate SE. **a** Stimulation of AR formation by soil contact. Plants were artificially fallen down, as described in Fig. 3b. The leaf nodes of the fallen plants were then embedded in the soil for 10 days. Representative plants were photographed (left photographs). Arrows and arrowheads indicate ARs formed at the lower and upper sides of the fallen tillers, respectively. Asterisks mark AR primordia. AR emergence was statistically analyzed (right graph). **b** Induction of AR formation by sand-driven mechanical touch. Leaf nodes were covered with soil or sand stack (left photographs). To minimize the sand humidity, miracloth was put in between the soil and sand stacks (sand+paper). AR emergence was statistically analyzed (right graph)

To test the hypothesis that direct contact of the leaf nodes with soil particles is a prerequisite for the induction of AR formation, plants were artificially fallen down, and the leaf node parts were completely embedded in the soil. Surprisingly, the soil-embedded leaf nodes produced multiple ARs on both the lower and upper sides, unlike wind-driven fallen plants that produced ARs mostly on the lower, soil-contacting side of the leaf nodes (Fig. 4a).

Next, the soil underneath the leaf nodes were removed so that these plant organs are not in direct contact with soil particles during wind treatments (Additional file 6: Fig. S6a). Notably, the frequency of AR emergence was comparable to that observed in plants grown under normal conditions (Additional file 6: Fig. S6b). It is therefore evident that the direct contact of the leaf nodes with soil particles in fallen plants, not the physical bending of the mesocotyls itself, is critical for the induction of AR formation.

Natural soil contains various nutrients in addition to water. It has been reported that soil nutrients affect AR emergence and elongation at the leaf nodes in *Brachypodium* [24]. In addition, waterlogging is a well-known stress that induces AR emergence in cereal crops [25–27]. To examine whether soil nutrients or water affect AR formation on the leaf nodes, the first leaf nodes were covered with the soil but without the bending of the mesocotyls (Additional file 7: Fig. S7a). As inferred from our data on the necessity of the direct contact of the leaf nodes with soil particles for AR formation, the soil-covered plants formed ARs around the leaf nodes (Fig. 4b), further supporting the notion that physical contact with soil particles is sufficient for the induction of AR emergence from the leaf nodes.

Stacking of pure sand particles, which would eliminate or greatly reduce the effects of soil nutrients, around the leaf nodes still triggered the induction of AR formation from the leaf nodes with the frequency comparable to that observed in the soil-covered plants (Fig. 4b). Meanwhile, we put two layers of miracloth in between the sand stack and the soil layer to get rid of the majority of water flow from the soil layer to the sand stack (Additional file 7: Fig. S7b; Additional file 8: Fig. S8). Again, the frequency of AR emergence was comparable to those observed in plants covered with the soil layer or the sand stack (Fig. 4b). Together, these observations unequivocally demonstrate that physical contact of the leaf nodes with soil particles, but neither soil nutrients nor water logging, provokes AR emergence.

### Auxin mediates the wind-mediated mechano-stimulation of AR emergence

Auxin is one of the key growth hormones that mediate root morphogenesis [28–30]. Ethylene is another growth hormone that is known to play a role in the induction of AR emergence under stressful conditions, as observed with submerged plants in flooded areas [25, 26]. Our observations indicate that ARs form mostly at the leaf nodes that are directly contacted with soil particles.

**Fig. 5:**
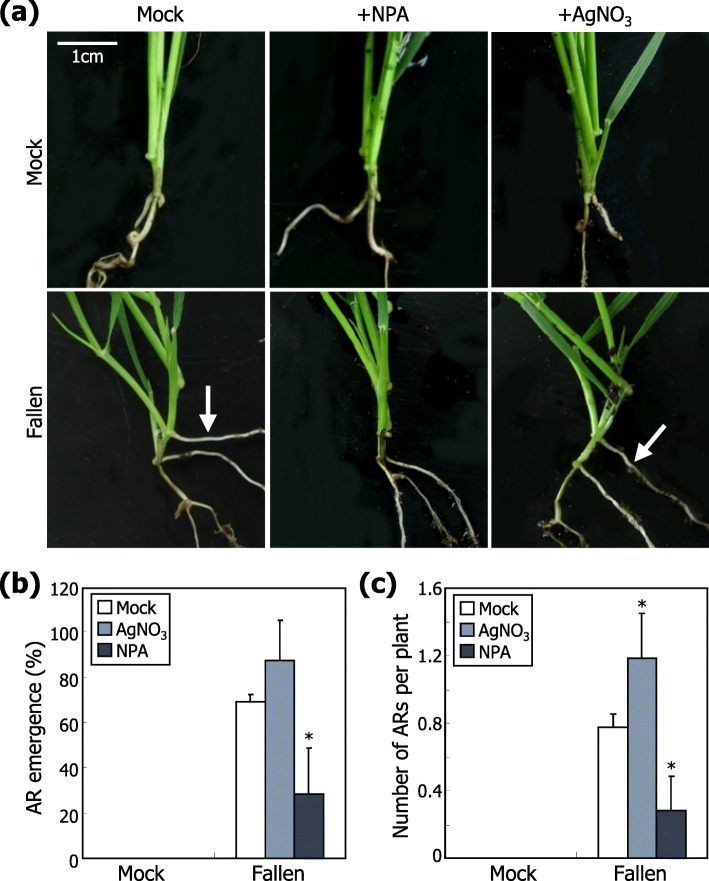
Auxin-mediated induction of AR formation. To examine the effects of growth hormone inhibitors on AR formation, three-week-old plants grown in soil were artificially fallen down, and the NPA or AgNO_3_ solution (1 μM each) was sprayed once a day for 10 days. **a** Representative plants were photographed. Arrows indicate ARs. AR emergence (**b**) and number of ARs per plant (**c**) were analyzed. Three independent experiments, each consisting of 16 plants, were statistically analyzed (*t*-test, *P < 0.01). Error bars indicate SE

To obtain insights into how mechanical stimulation by direct contact with soil particles triggers the induction of AR emergence, we employed chemicals that specifically alter auxin or ethylene functioning. Plant shoots were treated with either an auxin transport inhibitor, *N*-1-Naphthylphthalamic acid (NPA), or an ethylene perception inhibitor, AgNO_3_ [31, 32]. The inhibitor-treated plants were then artificially fallen down using arresting wires. It was found that application of NPA significantly reduced the frequency of AR emergence, while application of AgNO3 slightly increased the incidence of ARs (Fig. 5). We verified that exogenous application of 1 μM NPA was sufficient to inhibit auxin transport using the *Brachypodium* DII-VENUS reporter plants (Additional file 9: Fig. S9). In addition, exogenous application of IAA to fallen plants promoted AR formation (Additional file 10: Fig. S10). Meanwhile, exogenous application of 1 mM or higher concentrations of AgNO_3_ showed a slight increase of AR emergence (Additional file 11: Fig. S11), similar to what observed with exogenous application of 1 μM AgNO_3_. These observations indicate that auxin plays a major role in mediating the mechano-stimulation of AR formation in *Brachypodium*.

### *Brachypodium WOX* and *LBD* genes are auxin-responsive

In *Arabidopsis*, while AR formation does not occur under normal growth and developmental conditions, the plant-specific WOX transcription factors, such as AtWOX11 and AtWOX12, function as key regulators of AR development during organogenesis [33]. Similarly, a rice homolog of the WOX members, OsWOX11, plays a role in the initiation and development of crown roots [22].

**Fig. 6:**
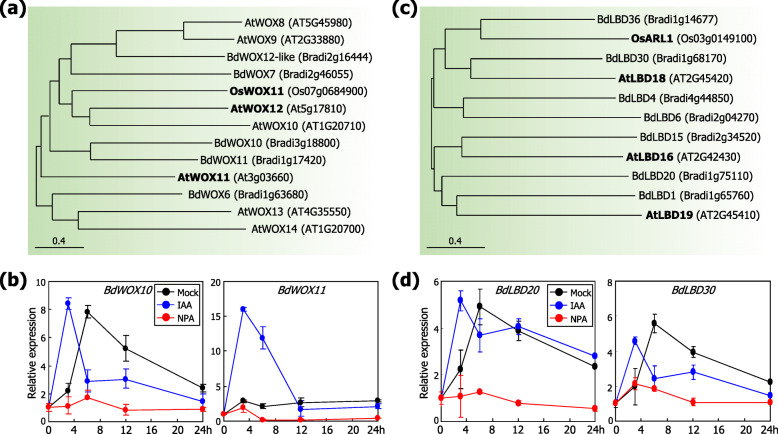
Auxin-mediated stimulation of *WOX* and *LBD* gene expression in artificially fallen plants. The phylogenetic trees were generated using the Neighbor-Joining method of the MEGA7 software (https://www.megasoftware.net) (**a, b**). In the phylogenetic analysis, protein members that have been functionally characterized were marked in bold. For gene expression analysis, three-week-old plants grown in soil were sprayed with either 0.1 mM IAA or 1 μM NPA solution and then artificially fallen down to the soil surface (**c, d**). The first leaf nodes and their internodes were harvested at the indicated time points for the extraction of total RNA samples. Transcript levels were analyzed by RT-qPCR. Biological triplicates, each consisting of 15 plants, were statistically analyzed. Error bars indicate SE. **a** Phylogenetic analysis of *Brachypodium* WOX proteins. **b** Effects of auxin and NPA on the transcription of *Brachypodium WOX* genes. **c** Phylogenetic analysis of *Brachypodium* LBD proteins. **d** Effects of auxin and NPA on the transcription of *LBD* genes

Phylogenetic analysis revealed that there are a group of potential WOX proteins in the *Brachypodium* genome (Fig. 6a). Amino acid sequence analysis revealed that multiple *Brachypodium* WOX proteins and those identified from *Arabidopsis* and rice, such as AtWOX11, AtWOX12, and OsWOX11, belong to a common cluster. On the basis of the notion that auxin is a key regulator of the wind-induced AR formation in *Brachypodium*, we examined whether *BdWOX* genes are responsive to auxin. Plants were sprayed with indole-3-acetic acid (IAA) or NPA solution and subsequently fallen down artificially. The first leaf nodes and their internodes were harvested for gene expression assays. It was found that the transcription of the *BdWOX10* and *BdWOX11* genes was induced by more than 10-fold by exogenous auxin application at earlier time points following auxin application but drastically suppressed to a basal level by NPA (Fig. 6b).

ARL1 is a rice homolog of the LBD transcription factors [21]. It has been shown that rice mutants lacking the *ARL1* gene is not able to induce AR formation [21]. It has been also reported that a few *Arabidopsis* LBD members, such as AtLBD16, AtLBD18, and AtLBD19, play crucial roles in the formation of lateral roots [34, 35]. Phylogenetic analysis revealed that multiple BdLBD proteins, especially BdLBD20 and BdLBD30, are homologous to AtLBDs and OsARL1 (Fig. 6c). Gene expression assays showed that the transcription of the *BdLBD20* and *BdLBD30* genes was significantly induced by exogenous auxin application but suppressed by NPA (Fig. 6d), similar to the effects of auxin and NPA on the *BdWOX* gene expression. These observation suggest that the BdWOX and BdLBD proteins are involved in auxin signaling during the induction of AR formation in *Brachypodium*.

### *WOX* and *LBD* genes are induced by the wind-mediated mechano-stimulation

**Fig. 7:**
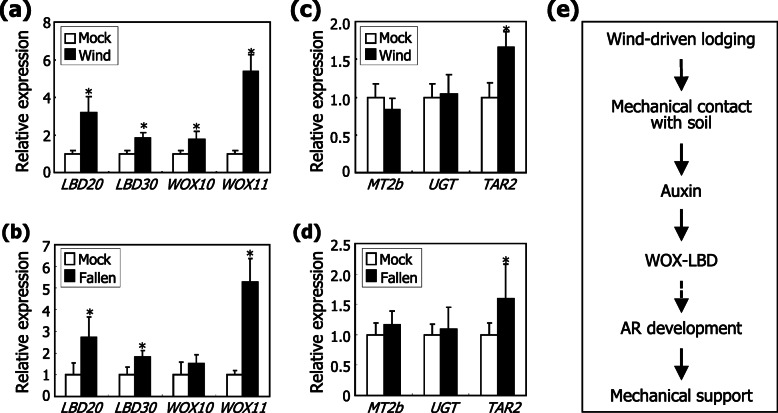
Induction of *WOX* and *LBD* genes by wind-mediated mechanical stimulation. Following stimulation by wind and artificial falling down, the first leaf nodes and their internodes were harvested for total RNA extraction, and transcript levels were analyzed by RT-qPCR. Biological triplicates, each consisting of 15 plants, were statistically analyzed (*t*-test, *P < 0.01). Error bars indicate SE. **a, b** Transcription of *WOX* and *LBD* genes. Three-week-old plants grown in soil were exposed to either a constant wind flow (**a**) or artificially fallen down (**b**) for 6 h. **c, d** Transcription of ethylene response genes. The plants treated with either wind flow (**c**) or falling down (**d**) for 6 h were used. **e** Schematic model of auxin-mediated AR formation under wind-induced lodging stress conditions. In response to wind flow, plants falls down, imposing a mechanical stimuli on the leaf nodes. The mechanical stimulation would induce the auxin-dependent expression of *WOX* and *LBD* genes. We propose that the WOX/LBD-mediated auxin signals trigger the initiation and development of ARs, leading to plant adaptation to lodging stress

A last question was whether the BdWOX and BdLBD proteins are linked with the wind-induced mechano-stimulation of AR formation in *Brachypodium*. We examined whether the expression of the *BdWOX* and *BdLBD* genes is altered in response to wind and mechanical stimuli. Gene expression assays showed that the auxin responsiveness of their transcriptions reaches the peak 6 h following auxin treatments (Fig. 6). Therefore, plant materials were harvested 6 h following wind or mechanical treatments. As expected from the notion that the *WOX* and *LBD* genes are involved in lateral root development or AR formation in rice and *Arabidopsis* [21, 22, 33–35], the transcription of *BdWOX* and *BdLBD* genes was markedly induced by wind treatments and artificial falling down (Fig. 7a, b). The gene induction was also observed in the soil-covered leaf nodes (Additional file 12: Fig. S12), supporting that the BdWOX and BdLBD transcription factors are likely to be involved in the wind-mediated mechano-stimulation of AR formation in *Brachypodium*.

Genes encoding UDP-glycosyltransferase 76C2-like (UGT76–4) and tryptophan aminotransferase-related protein 2-like (TAR2) have been identified as ethylene response markers in *Brachypodium* [36]. Metallothionein2b (MT2b) is a potent scavenger of reactive oxygen species (ROS), which induces cell death during AR emergence [19]. Gene expression assays revealed that the transcription of *BdUGT76–4* and *BdMT2b* genes was not discernibly affected by wind treatments or artificial falling down of the leaf nodes (Fig. 7c, d). In addition, auxin and NPA treatments did not discernibly affect the transcription of the genes (Additional file 13: Fig. S13). Overall, it is evident that auxin plays a primary role in the mechano-stimulation of AR formation possibly by modulating the expression of *BdWOX* and *BdLBD* genes, which is in accordance with the effects of auxin inhibitors on AR formation (Fig. 5). In contrast, ethylene does not seem to play a direct role in this thigmomorphogenic process.

Meanwhile, the transcription of *BdTAR2* gene was slightly induced in wind-treated and artificially fallen plants (Fig. 7c, d). It is anticipated that TAR2 is involved in the reprogramming process of root architecture under wind conditions in *Brachypodium*, as has been suggested in *Arabidopsis* [37].

Altogether, our findings illustrate a distinct auxin signaling pathway that mediates the wind-induced mechano-stimulation of AR formation in *Brachypodium* (Fig. 7e). Under extreme wind conditions, plants fall down, resulting in the direct contact of the leaf nodes with soil particles. The physical contact imposed on the leaf nodes induces the auxin-mediated expression of *BdWOX* and *BdLBD* genes, possibly leading to the induction of AR emergence. We propose that the auxin-mediated mechano-stimulation of AR development serves as an adaptive strategy, by which plants sustain their normal growth and productivity in extreme wind areas.

## Discussion

### AR as an adaptive developmental device under wind-induced mechanical stress

ARs are developmentally distinct from primary and lateral roots in that they are derived mostly from nonroot tissues, such as root-shoot junctions and stem nodes. They occur through both normal developmental processes and stress response pathways prominently in grasses and cereal crops [18]. Not only the economic and ecological values of ARs but also their importance as food source are becoming apparent over the past decades. Economically, cut AR parts are capable of producing new individuals and thus frequently used in horticulture industries. Moreover, ARs play critical roles in plant adaptive processes under changing environments, underscoring their economic values in agricultural industry.

It is well-known that ARs help plants survive under certain abiotic and biotic stress conditions, such as flooding and nutrient deficiency, which are frequently encountered in nature [38, 39] The most extensively studied is submergence of plants in agricultural and natural ecosystems, often accompanying oxygen deficiency in plants. Rice is a semiaquatic plant that readily generates ARs upon flooding, and molecular signaling events leading to the stimulation of AR formation during flooding have been intensively studied [25–27]. Submergence induces ethylene biosynthesis, and the gaseous growth hormone is trapped by water barrier. The accumulated ethylene triggers the production of ROS, which, in conjunction with ethylene, triggers epidermal programmed cell death for the induction of AR emergence [19].

The initiation and development of ARs vary widely, depending on the types of nutrients and stress and root types. For example, the density of lateral roots originated in the pericyclic cells of crown roots increases when exposed to locally high concentrations of nitrate, while those originated from seminal roots are unaffected under similar nutrient conditions in maize [39, 40]. It is known that various root types that are formed in different soil depths have differential efficiency of nutrient uptakes because nutrients are frequently distributed unevenly in relation to the soil layers. One typical example is uptake of phosphorus, which is available primarily in the soil surface layer. Therefore, while an increased number of surface roots enhances tolerance to phosphorus deficiency, deeper roots only poorly respond to phosphorus-deficient soil [41]. These phenomena highlight the complex regulation of AR emergence under diverse nutrient-deficient conditions.

### Hormonal regulation of post-embryonic root formation

AR organogenesis is modulated via a coordinated interaction of various hormonal signaling networks. Generally, auxin plays a primary role during the post-embryonic root formation [29]. In *Arabidopsis*, it is well-established that auxin controls lateral root formation [29, 42]. Auxin also plays essential roles throughout the initiation and elongation of lateral roots by modulating auxin-responsive transcription factors and trafficking of PIN-FORMED (PIN) auxin transporters [30]. In rice, it is proven that auxin flow through the PIN transporters is critical for the induction of AR emergence [43].

Other growth hormones, such as cytokinins, strigolactones, brassinosteroids, JA, ABA, and GA, are also involved in the induction of AR formation [44]. For example, auxin signals activate the expression of genes encoding Gretchen Hagen 3-like proteins, which sustain JA homeostasis during AR formation [45]. It is known that cytokinin and auxin function antagonistically in regulating AR formation [46]. Meanwhile, ABA inhibits GA and ethylene signaling in the course of AR emergence and elongation [25].

In this study, we demonstrated that *Brachypodium* efficiently adapts to wind stress, in which wind-induced falling down of the shoots promotes AR formation via a WOX/LBD-mediated auxin signaling pathway. Phylogenetic analysis, gene expression studies, and transcriptional responses to mechanical stimuli identified a subset of *Brachypodium WOX*/*LBD* genes that are likely to be involved in the auxin-mediated mechanical stress adaptation. These genes are expressed mainly in the leaf nodes, from which ARs are formed. Their gene transcription is induced upon exposure to wind and mechanical falling down of the leaf nodes. Interestingly. It has been recently shown that JA interacts with auxin in regulating AR formation under stress conditions in *Arabidopsis* [47]. On the basis of the roles of JA in touch responses [48], it is possible that the JA-auxin signaling crosstalks would also module the AR formation in *Brachypodium*.

It was found that the induction of *WOX*/*LBD* genes did not occur in NPA-treated plants, indicating that auxin transport is important for the WOX/LBD-mediated mechanical adaptation process. It is currently unclear how mechanical stimuli are linked with auxin signaling at the molecular level. It is possible that both auxin transport and biosynthesis, and perhaps auxin sensitivity as well, would be involved in the wind-induced mechanical stress responses. Since extended treatments with NPA may trigger other developmental processes, such as crown root, lateral root, and primary root growth, direct measurements of endogenous auxin contents will help clarify its roles during the touch-induced AR formation. Functional identification of auxin biosynthetic enzymes and wind/mechanical stimuli-responsive PIN proteins and direct measurements of endogenous auxin contents under mechanical stress conditions would help elucidate the underlying molecular and biochemical mechanisms.

The *TAR2* gene, an ethylene response marker gene [36], was induced slightly by wind exposure. Meanwhile, NPA treatments decreased its transcription, while auxin does not have any effects on the gene transcription. The *Arabidopsis TAR2* gene encodes a tryptophan aminotransferase that mediates auxin biosynthesis in response to ethylene signaling [49]. It has been reported that the *Arabidopsis TAR2* gene is required for the emergence of lateral roots under low nitrogen stress conditions [37]. It is envisioned that TAR2-mediated ethylene signals do not play a direct role in the wind-induced thigmomorphogenic adaptation process but instead affect indirectly the AR formation by affecting auxin biosynthesis.

In accordance with the seemingly limited role of the ethylene response markers, UGT76–4 and TAR2, assays with ethylene perception inhibitor showed that wind-induced AR formation is not discernibly affected by ethylene signaling. Treatments with the ethylene perception inhibitor AgNO_3_ only slightly induces AR emergence. However, the chemical treatments do not exhibit any dosage effects on the incidence of AR emergence. It is thus postulated that the marginal effects of AgNO_3_ on AR formation might be caused by a side effect of the chemical during plant morphogenesis [50]. Nevertheless, it is still possible that ethylene plays a certain role in the thigmomorphogenic AR formation through as-yet unidentified signaling crosstalks with auxin. Further works using *Brachypodium* mutants having defects in auxin and ethylene biosynthesis or signaling and genome-wide gene expression studies would be of great helps to explore the possibility.

### Induction of AR development by wind-mediated mechanical stimuli

Mechano-stimulation of AR formation has been characterized in rice [51]. Ethylene promotes ROS accumulation by suppressing the function of the ROS scavenger MT2b. External or internal mechanical pressures simultaneously promote epidermal cell death, which facilitates the emergence of ARs. The two mutually collaborative signals provide an elaborative spatiotemporal information to initiate AR formation in appropriate nonroot tissues in rice.

Our findings showed that wind-mediated falling down does not affect the expression of the *Brachypodium MT2b* gene, indicating that the wind-mediated thermomorphogenic AR development in *Brachypodium* somewhat differs from the ROS-mediated mechanical stimulation of AR emergence in rice. A critical issue is as to cellular receptors or sensory molecules that are capable of perceiving mechanical signals. One such potential candidate is cytosolic Ca^2+^ ion, a ubiquitously conserved signaling component in all living organisms [52, 53]. It has been observed that mechanical perturbations are immediately followed by rapid increase in cytosolic Ca^2+^ concentrations in a dose-dependent manner in *Nicotiana plumbaginifolia* [54]. The *Arabidopsis* and rice genomes possess ten and five mechano-sensitive Ca^2+^ ion channels, respectively, supporting the involvement of Ca^2+^ ion as a sensing molecule or second messenger in the mechano-adaptation process [55]. Intriguingly, it is known that the Ca^2+^-permeable mechano-sensitive channel 1 (MCA1) mediates Ca^2+^ uptake in response to agar hardness on culture media in *Arabidopsis* [56]. Overall, it is now apparent that thigmomorphogenic response is a critical adaptation process to cope with mechano-disturbing environmental stresses, while underlying signaling schemes and molecular mechanisms are to be investigated in the future.

There is a steadily increasing concern about wind-induced damages on plant ecosystems and crop productivity, and thus further understanding molecular mechanisms underlying the wind-induced thigmomorphogenic adaptation is an important issue in the field. Under these circumstances, our findings would contribute to further elucidating the molecular signaling cascades that mediate AR development, which are readily applicable to developing mechano-resistant crops.

## Conclusion

Here, we demonstrate that *Brachypodium distachyon*, a model grass widely used for studies on bioenergy crops and cereals, efficiently adapts to wind-driven mechanical stress by inducing AR formation via auxin signaling. We found that not the bending of the coleoptiles itself but their direct contact with soil particles triggers the transcriptional induction of a group of auxin-responsive genes encoding WOX and LBD proteins, which are likely to be associated with AR formation.

## Methods

### Plant materials and growth conditions

*Brachypodium distachyon* ecotype Bd21–3, which is a community standard diploid inbred line, was obtained from Dr. John P. Vogel and used in all experiments. The *Brachypodium proZmUbi:DII-VENUS* transgenic seeds were obtained from Dr. Devin Lee O’Connor. *Brachypodium* plants were grown in a controlled growth chamber with relative humidity of 60% under long day conditions (16-h light/8-h dark). Growth conditions were set at 23 °C with white light illumination provided by FLR40D/A fluorescent tubes (150 μmol photons/m^2^s, Osram, Seoul, Korea).

### Wind treatment

An electronic fan (EF-73HK, Hanil, Korea) was used to generate wind force, and the speed of wind flow was measured using an anemometer (ST-112, Sincon, Korea). For wind acclimation analysis, three-week-old plants grown in soil were first exposed to a constant unidirectional wind flow (2.8 m/s) for 10 days. A relatively low speed of wind was used to induce a wind acclimation process during the pretreatment. The wind-acclimated plants were then subjected to wind flow and photographed. Wind sensitivity was analyzed by measuring the angles of lodged tillers relative to the soil surface. All experiments were repeated three times for statistical analysis, unless otherwise mentioned.

Temperature and humidity of air were measured using an USB data logger (SL170, SELCO, Denmark). The moisture status of soil was measured using a soil moisture meter (0101HHEHAY, HUIXUAN, China). Thermal images of plants were recorded using an infrared camera (T420, FLIR, USA), and the images were analyzed using the FLIR software (http://www.flirkorea.com/home/).

To examine the role of AR in lodging tolerance, three-week-old plants grown in soil were exposed to a constant unidirectional wind flow for 10 days. The plants harboring ARs were identified, and visible ARs were artificially removed. The AR-removed or -retaining plants were subjected to wind flow, and the tiller angles were measured.

### Falling down and gravity

To examine the effects of falling down on the induction of AR formation, three-week-old plants grown in soil were artificially fallen down by bending the mesocotyls just below the coleoptile nodes using arresting wires so that the leaf nodes directly touch soil particles. Arresting wires were carefully equipped not to touch the leaf node parts, which would potentially trigger a touch-induced thigmomorphogenic response. Ten days following the falling down treatment, the frequency of AR emergence was measured, in which only ARs of longer than 5 mm in length were counted. All experiments were repeated three times for statistical analysis, unless otherwise mentioned.

For gravi-stimulation assays, plants were rotated by 75 degrees and further grown for 10 days. To maintain the horizontally positioned stems and leaf nodes, plants were equipped with supporting wires. For assays on the effects of falling down in vertically positioned plants, artificially fallen plants were rotated to position vertically the stems and leaf nodes. One side of the leaf nodes was allowed to be in direct contact with soil particles. The plants were further grown for 10 days before measuring AR emergence.

### Mechanical contact

To examine the effects of mechanical touch on the induction of AR emergence, the first leaf nodes of three-week-old plants was completely covered with soil or sand layer. In addition, two layers of miracloth (Merck, Germany) were positioned in between the sand stack and the grounding soil layer to keep the sand stack in a semidry state. To investigate the effects of falling down without soil contact, plants were fallen down, and the soil beneath the leaf nodes was carefully removed so that soil particles do not touch the leaf nodes. The plants were grown for 10 days before measuring AR emergence.

### Measurement of water content

Three-week-old plants grown in soil were exposed to wind flow for 10 days. The fresh leaves were harvested and weighed before dried at 65 °C for 2 days. The dried leaves were weighed, and the water content was calculated according to the following formula: water content (%) = (fresh weight - dry weight)/fresh weight × 100. All experiments were repeated three times for statistical analysis, unless otherwise mentioned.

### Chemical treatments

The auxin transport inhibitor NPA (Sigma, USA), the ethylene perception inhibitor AgNO_3_ (Sigma, USA), and the auxin IAA (Sigma, USA) solutions were prepared in Tween 20 (Amresco, USA). The NPA (1 μM in 0.05% (v/v) aqueous Tween 20), AgNO_3_ (1 μM or 100 μM in 0.5% (v/v) aqueous Tween 20) solution, IAA (10 μM or 10 μM in 0.5% (v/v) aqueous Tween 20) or 0.5% (v/v) aqueous Tween 20 alone was sprayed once a day for 10 days onto three-week-old plants grown in soil, each spray using approximately 1 ml per plant. Following the first spray, plants were artificially fallen down and further grown for 10 days before analysis. All experiments were repeated three times for statistical analysis, unless otherwise mentioned.

To verify the effects of NPA on auxin accumulation in *Brachypodium*, the *Brachypodium proZmUbi:DII-VENUS* transgenic plants, which have been successfully employed to monitor auxin distribution in this plant species [57], were analyzed. The reporter plants were fallen down and further grown for 2 days to induce AR formation. A solution of 1 μM NPA was sprayed on the fallen plants and incubated for 6 h. AR primordia-forming nodes were harvested and sectioned using razor blade. The sectioned nodes were laid on slide glass and subjected to fluorescence imaging using an Olympus BX53 microscope. The following laser and filter setup were used: Olympus U-HGLGPS laser, 488 nm for excitation, 498 to 544 nm for emission to detect VENUS signals. The magnification value was set to 4. Confocal images were also obtained using a LSM710 laser scanning confocal microscope (ZEISS, Germany) under identical microscopic setting.

For gene expression assays, the NPA (1 μM in 0.05% (v/v) aqueous Tween 20) or IAA (100 μM in 0.5% (v/v) aqueous Tween 20) solution was sprayed onto three-week-old plants, which were subsequently fallen down. The first leaf nodes and their internodes were harvested at 0, 3, 6, 12, and 24 h following chemical treatments for the extraction of total RNA samples.

### Phylogenetic analysis

The amino acid sequences of the WOX proteins from *Brachypodium* (BdWOX6, BdWOX7, BdWOX10, BdWOX11, and BdWOX12-like), *Arabidopsis* (AtWOX8, AtWOX9, AtWOX10, AtWOX11, AtWOX12, AtWOX13, and AtWOX14), and rice (OsWOX11) were obtained from the NCBI database (https://www.ncbi.nlm.nih.gov/pubmed). The amino acid sequences of the LBD proteins from *Brachypodium* (BdLBD1, BdLBD4, BdLBD6, BdLBD15, BdLBD20, BdLBD30, and BdLBD36), *Arabidopsis* (AtLBD16, AtLBD18, and AtLBD19), and rice (OsARL1) were similarly obtained from NCBI. Phylogenetic analysis was carried out using the Neighbor-Joining method (Bootstrap method, number of bootstrap replications: 500, Poisson model, rates among sites: uniform rates, gaps/missing data treatment: complete deletion) from MEGA 7 software (https://www.megasoftware.net/).

### Gene transcript analysis

Total RNA was extracted from appropriate plant materials using the RNeasy Plant Mini Kit according to the manufacturer’s procedure (Qiagen, USA). Reverse transcription-mediated quantitative PCR (RT-qPCR) was employed to analyze the levels of transcripts.

All RT-qPCR reactions was performed in 96-well blocks with the 7500 Real-Time PCR System (Applied Biosystems, USA) using the KAPA SYBR Green master mix (Sigma, USA) in a reaction volume of 20 μl. The two-step thermal cycling profile employed was 15 s at 95 °C for denaturation and 1 min at 60-65 °C, depending on the calculated melting temperatures of PCR primers, for annealing and polymerization. The *Brachypodium UBC18* gene (Bd4g00660) was included as internal control in the PCR reactions to normalize the variations in the amounts of primary cDNAs used. The PCR primers were designed using the Primer Express software installed in the system and listed in Additional file 14: Table S1.

### Statistical analysis

All experiments and analyses were conducted using multiple biologically independent samples, and the number of biological replicates is specified in each experiment. Statistical significance was determined using either one-way analysis of variance (ANOVA) with post hoc Tukey test (*P* < 0.01) or two-sided Student *t*-test with *P* values of < 0.01 for determining significant differences of more than three populations or pairwise comparisons, respectively.

## Supplementary information

**Additional file 1 Figure S1**. Acclimation of Brachypodium plants to wind stimulation.

**Additional file 2 Figure S2**. Effects of wind stimulation on the moisture contents of air and soil.

**Additional file 3 Figure S3**. Effects of wind flow on air and plant body temperatures.

**Additional file 4 Figure S4**. Physical dissection of wind stimulation.

**Additional file 5 Figure S5**. Experimental set-up for the lodging phenotypic analysis of Brachypodium plants against mechanical and gravity stimuli.

**Additional file 6 Figure S6**. Effect of falling down without direct soil contact on the induction of AR formation.

**Additional file 7 Figure S7**. Experimental set-up for the AR phenotypic analysis of plants against mechanical touch.

**Additional file 8 Figure S8**. Schematic diagram for measuring the moisture content of soil and sand layers.

**Additional file 9 Figure S9**. Fluorescent imaging of AR primordia in the DII-VENUS reporter plants.

**Additional file 10 Figure S10**. Effects of IAA on AR formation.

**Additional file 11 Figure S11**. Effects of ethylene perception inhibitor on the induction of AR formation.

**Additional file 12 Figure S12**. Induction of *BdWOX* and *BdLBD* genes by soil touch.

**Additional file 13 Figure S13**. Effects of auxin and NPA on the transcription of ethylene response genes.

**Additional file 14 Table S1**. Primers used.

## Data Availability

All data generated or analyzed during the current study are included in this published article and its supplementary information files. The raw data are available from the corresponding author on reasonable request following the publication of the work.

## Abbreviations

AR: Adventitious roots
LBD: LATERAL ORGAN BOUDARIES DOMAIN
ARL1: ADVENTITIOUS ROOTLESS1
WOX11: WUSCHE RELATED HOMEOBOX11
NPA: N-1-Naphthylphthalamic acid
UGT76–4: UDP-glycosyltransferase 76C2
TAR2: Tryptophan aminotransferase-related protein 2
Mt2b: Metallothionein2b
ROS: Reactive oxygen species
PIN: PIN-FORMED
JA: Jasmonic acid
ABA: Abscisic acid
GA: Gibberellic acid
MCA1: Mechano-sensitive channel
