# Supplementary material for: Auxin mediates the touch-induced mechanical stimulation of adventitious root formation under windy conditions in Brachypodium distachyon
Source: BMC Plant Biol. 2020 Jul 16;20:335. doi: 10.1186/s12870-020-02544-8 (PMC7364541; doi:10.1186/s12870-020-02544-8)
Supplement: Supplementary file 1 — Additional file 1 Figure S1. Acclimation of Brachypodium plants to wind stimulation. [file 12870_2020_2544_MOESM1_ESM.pdf]

Supplementary Figure 1

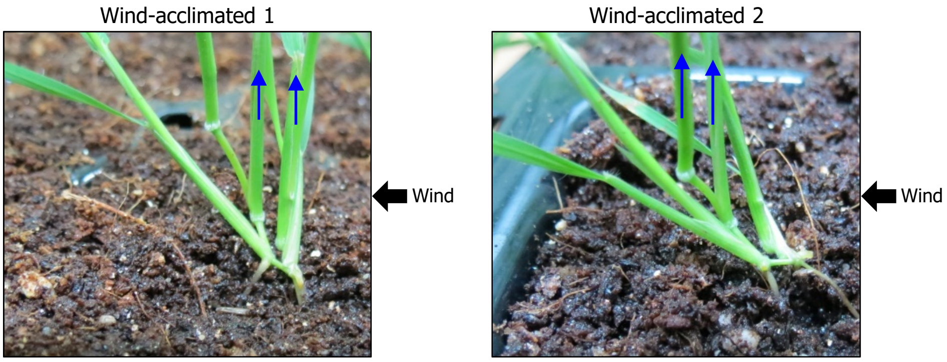

Fig. S1 Acclimation of *Brachypodium* plants to wind stimulation. Three-week-old plants grown in soil were exposed to a constant unidirectional wind flow for ten days. Enlarged views of two representative plants were displayed. Blue arrows indicate the vertical direction of the tillers recovered in the wind-acclimated plants.

Supplementary Figure 2

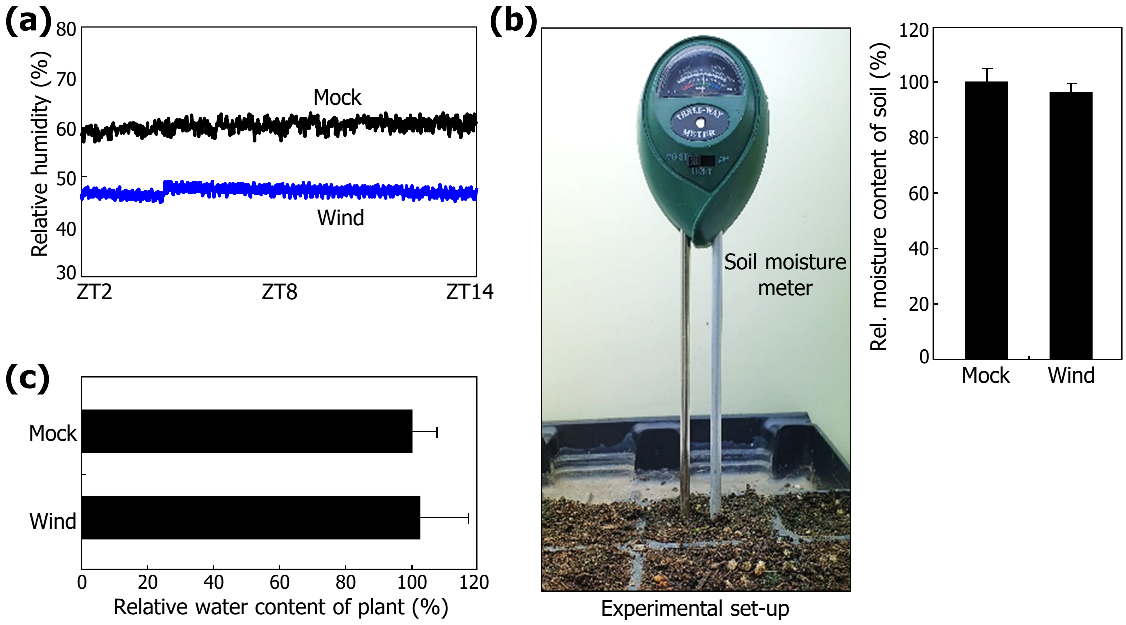

Fig. S2 Effects of wind stimulation on the moisture contents of air and soil. Experimental conditions for the measurements of air humidity and soil moisture are identical to those described in Fig. 2a. **a** Air humidity near the soil surface was recorded during the day. **b** Soil moisture was measured at a depth of 3 cm below the soil surface. Experimental set-up using a soil moisture meter was illustrated (left photograph), and relative soil moistures were measured (right graph). **c** Relative water contents of *Brachypodium* plants during wind treatments were also measured. Three-week-old plants were either grown under mock conditions or exposed to wind stimulation for ten days. Five independent measurements, each consisting of three leaves, were statistically analyzed (*t*-test, $*P < 0.01$). Error bars indicate standard error of the mean (SE).

Supplementary Figure 3

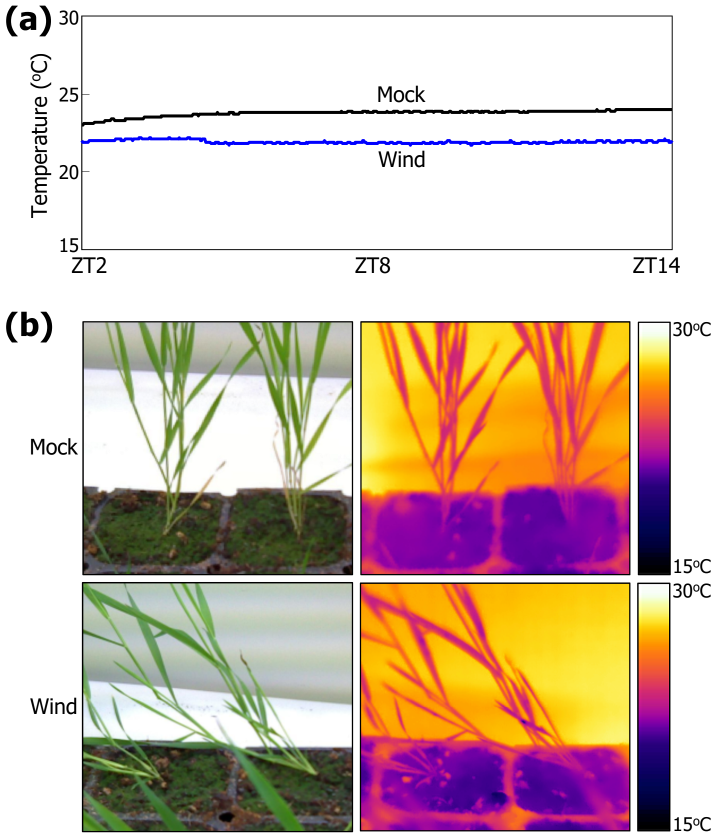

Fig. S3 Effects of wind flow on air and plant body temperatures. Three-week-old plants were either grown under mock conditions (no wind) or exposed to a unidirectional wind flow for varying durations. **a** Air temperatures. Air temperatures near the soil surface were measured during the day. ZT, zeitgeber time. Note that air temperatures under wind treatments are lower by approximately 2 °C compared to those under mock conditions. **b** Temperature of *Brachypodium* plants. Infrared thermographs of two representative plants were taken at ZT8. Note that plant temperatures were not discernibly altered in the wind-treated plants.

Supplementary Figure 4

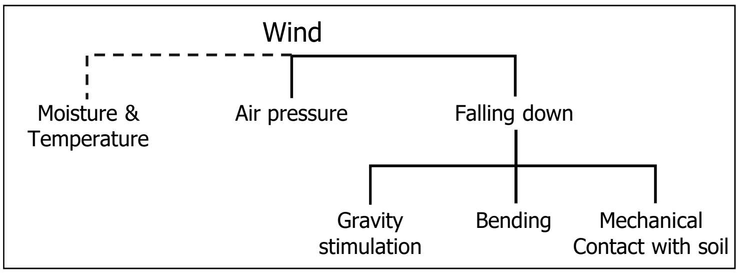

Fig. S4 Physical dissection of wind stimulation. Wind is a complicated mechanical stimulus that can be dissected into various effective factors. The potential effects of individual factors, such as air pressure, gravity stimulation, mechanical bending, and mechanical contact with the soil, on the induction of adventitious root (AR) formation were systematically assayed in this study. Note that air moisture and temperature are frequently affected by wind in nature. However, we found that these two factors are not significantly affected by wind flow in our assay conditions (marked by dashed line).

Supplementary Figure 5

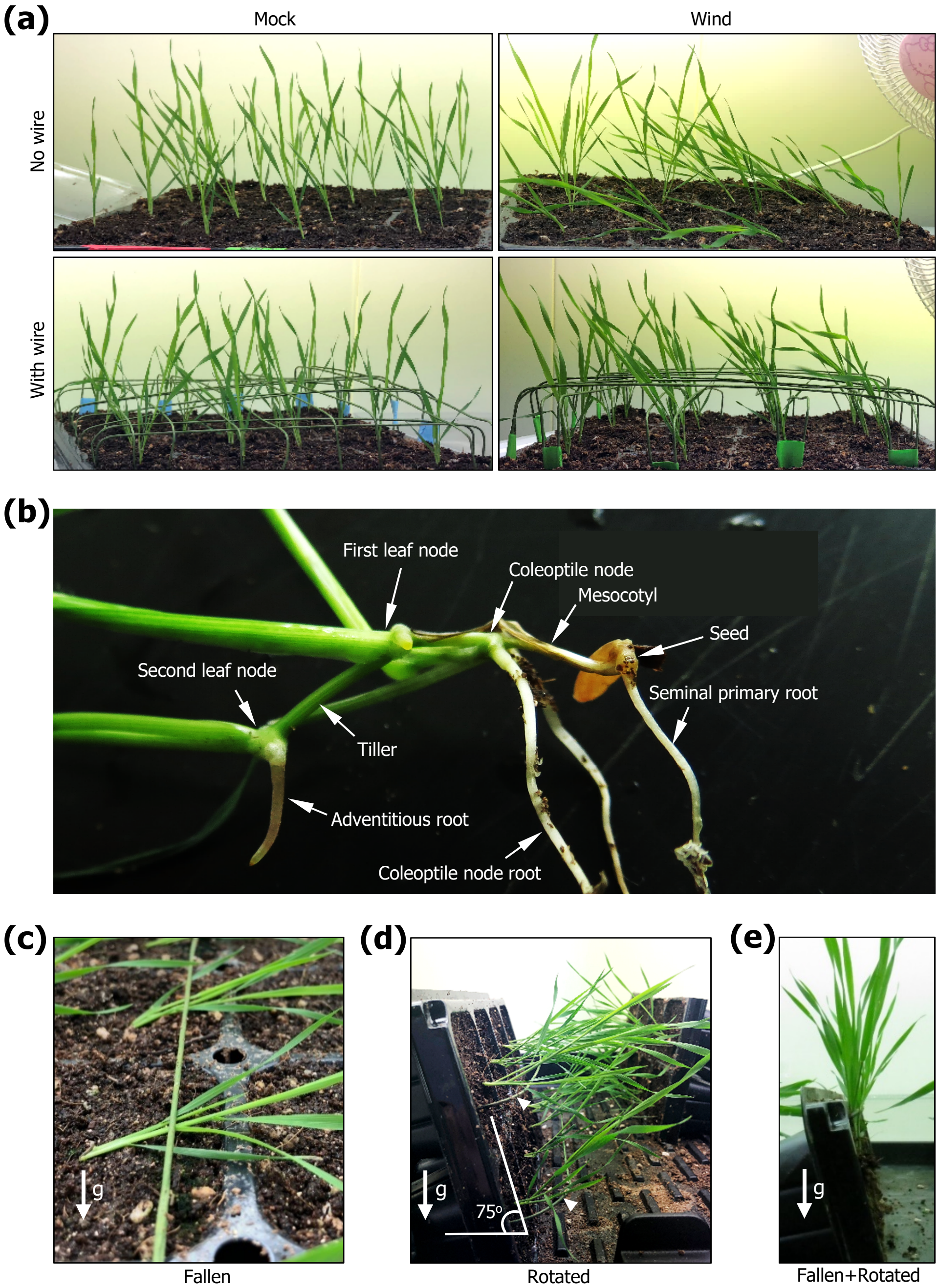

Fig. S5. Experimental set-up for the lodging phenotypic analysis of *Brachypodium* plants against mechanical and gravity stimuli. **a** Experimental set-up for Fig. 3a. **b** Detailed architecture of the *Brachypodium* root system. **c-e** Experimental set-up for 'fallen' and 'rotated' treatments. Plants were artificially fallen down to the soil surface by arresting wires (**c**, Fig. 3b). Plants were also gravi-stimulated by rotating horizontally (**d**, Fig. 3b). To prevent the shoots from falling downward, plants were supported by supporting wires (marked by arrowheads). The supporting wires were arranged carefully not to touch the leaf nodes. Artificially fallen plants were rotated by 75°, allowing them to grow upward (**e**, Fig. 3c). Note that only one side of the leaf nodes was physically contacted with soil particles.

Supplementary Figure 6

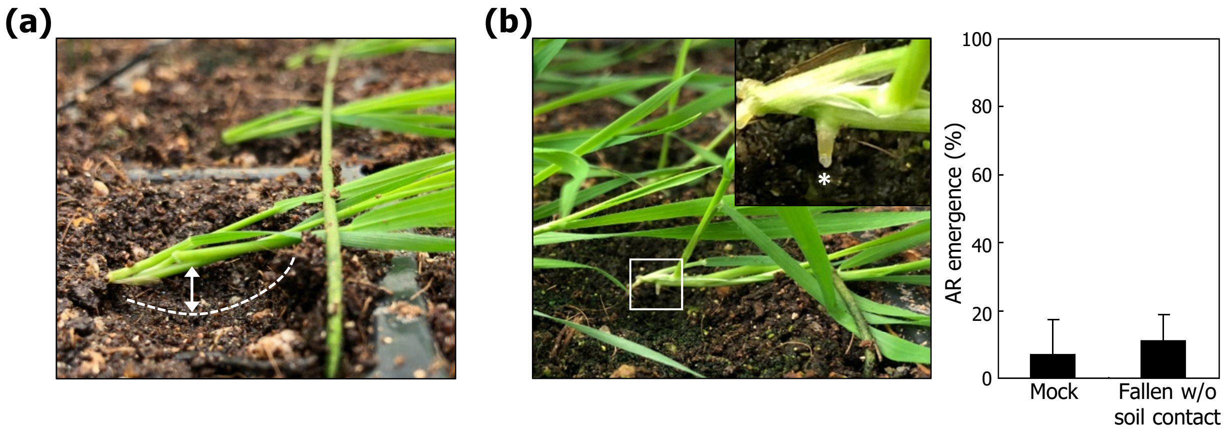

Fig. S6 Effect of falling down without direct soil contact on the induction of AR formation. Three-week-old plants grown in soil were assayed. Three measurements, each consisting of eight plants, were statistically analyzed. Error bars indicate SE. **a** Experimental set-up. Plants were artificially fallen down using arresting wires, and the soil around the leaf nodes were removed so that the leaf nodes does not directly touch the soil particles. **b** Effects of falling down without soil contact on AR emergence. The artificially fallen plants were incubated for ten days, and the number of AR emergence was counted. The inset indicates an enlarged view of the leaf node (marked by a white frame) (left photograph). Asterisk marks an AR primordium. Note that the number of ARs in artificially fallen plants without direct soil contact is similar to that in mock-treated plants (right graph).

Supplementary Figure 7

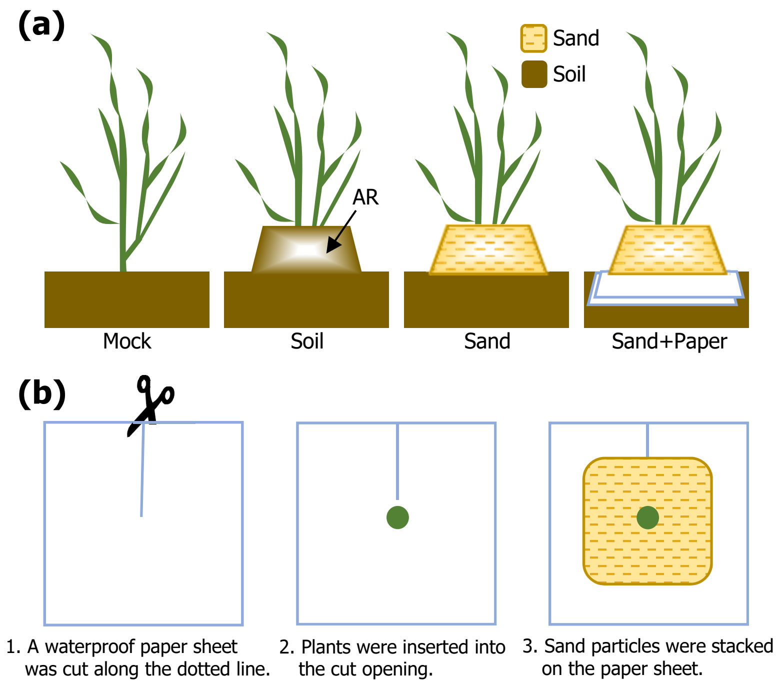

Fig. S7. Experimental set-up for the AR phenotypic analysis of plants against mechanical touch. **a** Experimental set-up for Fig. 4b. The leaf nodes of three-week-old plants grown in soil were covered with either soil or sand layer. In addition, to minimize the moisture of the sand layer, two layers of miracloth were positioned in between the sand and soil layers (sand+paper). It was found that the soil- or sand-covered leaf nodes produce similar numbers of ARs after ten days. **b** Detailed procedure for preparing the sand+paper set-up in **a**.

Supplementary Figure 8

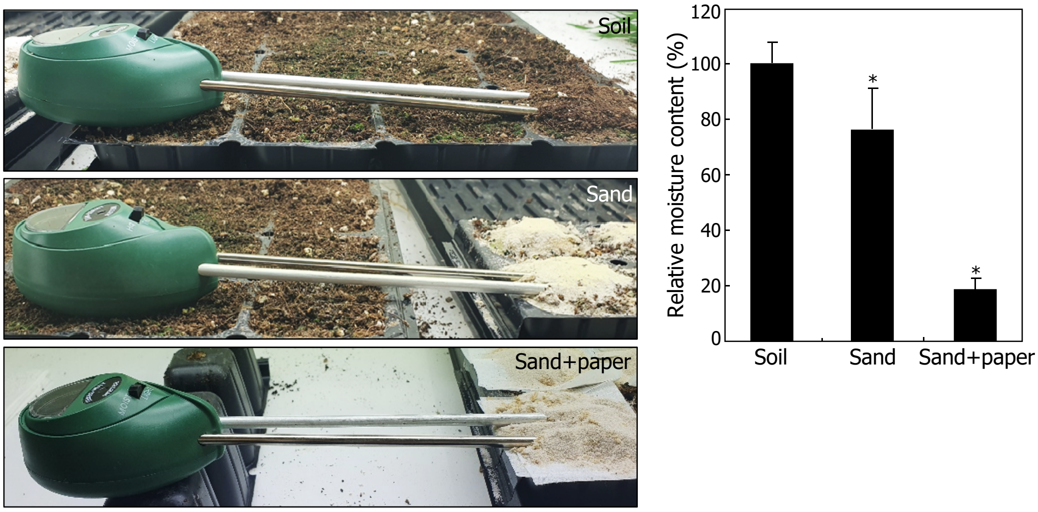

Fig. S8 Schematic diagram for measuring the moisture contents of soil and sand layers. Moisture contents were measured using a soil moisture meter under conditions identical to those described in Fig. 4b. Photographs of experimental set-up described in Fig. 4b were displayed. Three measurements, each consisting of five spots, were statistically analyzed (*t*-test, $^*P < 0.01$). Error bars indicate SE.

Supplementary Figure 9

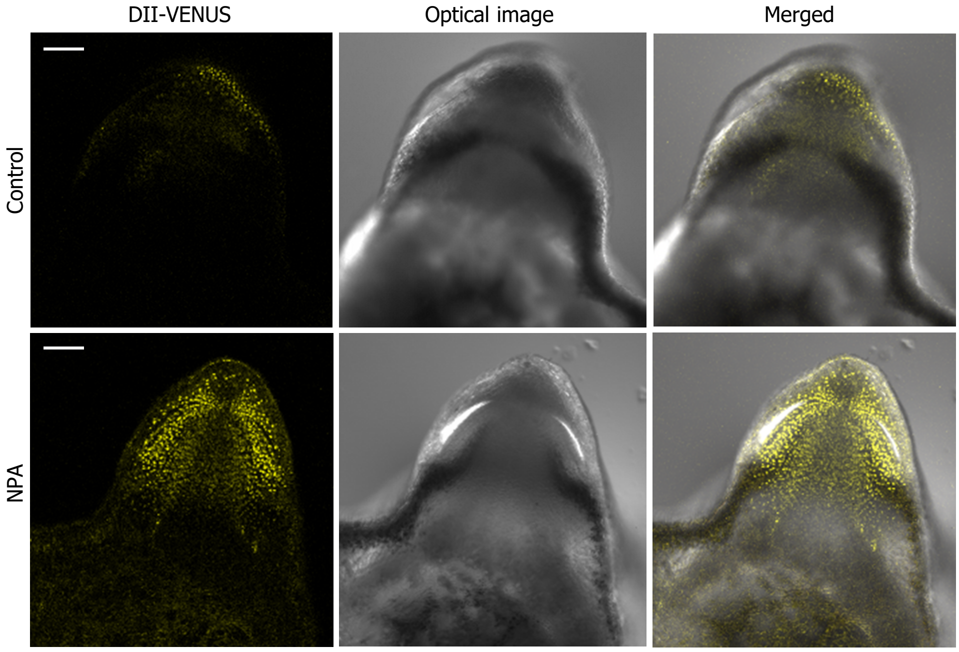

Fig. S9 Fluorescent imaging of AR primordia in the DII-VENUS reporter plants. The *Brachypodium* DII-VENUS reporter plants (*proZmUbi:DII-VENUS*) were employed to verify the negative effects of NPA on auxin accumulation in AR primordia under mechanical stress. The reporter plants have been successfully employed for the analysis of auxin accumulation in *Brachypodium*. Three-week-old reporter plants grown in soil were artificially fallen down, and a NPA solution (1 μM) was sprayed once a day for ten days. Fluorescent images of AR primordia that appeared from the leaf nodes were obtained. In this system, a lower fluorescent intensity represents a higher auxin accumulation, and vice versa. Scale bars, 100 μm.

Supplementary Figure 10

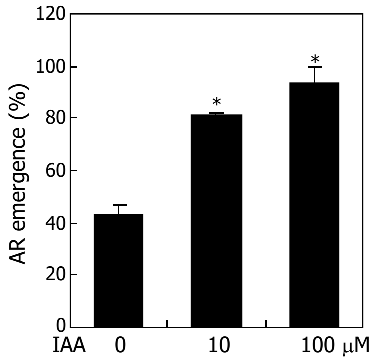

Fig. S10 Effects of IAA on AR formation. Three-week-old plants grown in soil were artificially fallen down, as described in Figure 5, and IAA solutions (either 10 or 100 μM) were sprayed once a day for ten days. Three measurements of AR emergence, each consisting of sixteen plants, were statistically analyzed (*t*-test, *$P < 0.01$). Error bars indicate SE.

Supplementary Figure 11

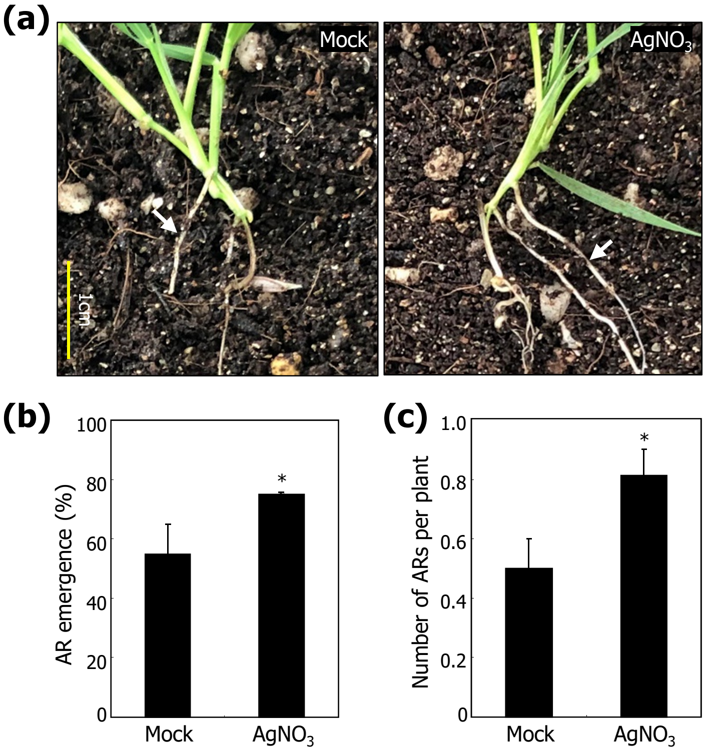

Fig. S11 Effects of ethylene perception inhibitor on the induction of AR formation. Three-week-old plants grown in soil were artificially fallen down, and a solution of 1 mM $AgNO_3$ was sprayed once a day for ten days. **a** Representative plants were photographed. White arrows indicate ARs. **b** AR emergence. **c** Number of ARs per plant. Three experiments, each consisting of sixteen plants, statistically analyzed (*t*-test, **P* < 0.01). Error bars indicate SE. Note the slightly promotive effects of $AgNO_3$ on the induction of AR formation, suggesting that ethylene is not a primary determinant of wind-mediated mechano-stimulation on the induction of AR formation.

Supplementary Figure 12

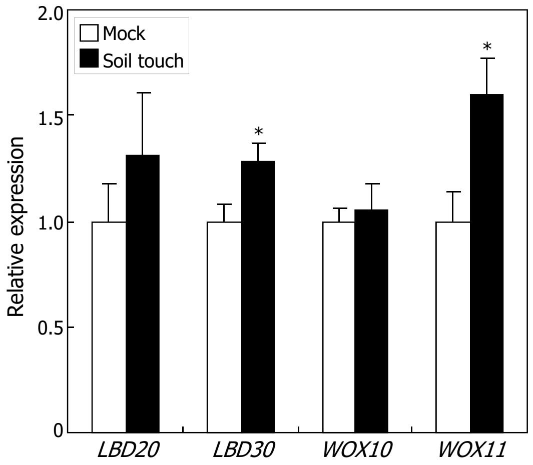

Fig. S12 Induction of *BdWOX* and *BdLBD* genes by soil touch. Following the mechanical stimulation by soil touch, the first leaf nodes and their internodes were harvested for total RNA extraction, and transcript levels were examined by reverse transcription-mediated quantitative PCR (RT-qPCR), as described in Fig. 6b. Biological triplicates, each consisting of eight independent plants, were statistically analyzed (*t*-test, *$P < 0.01$). Error bars indicate SE.

Supplementary Figure 13

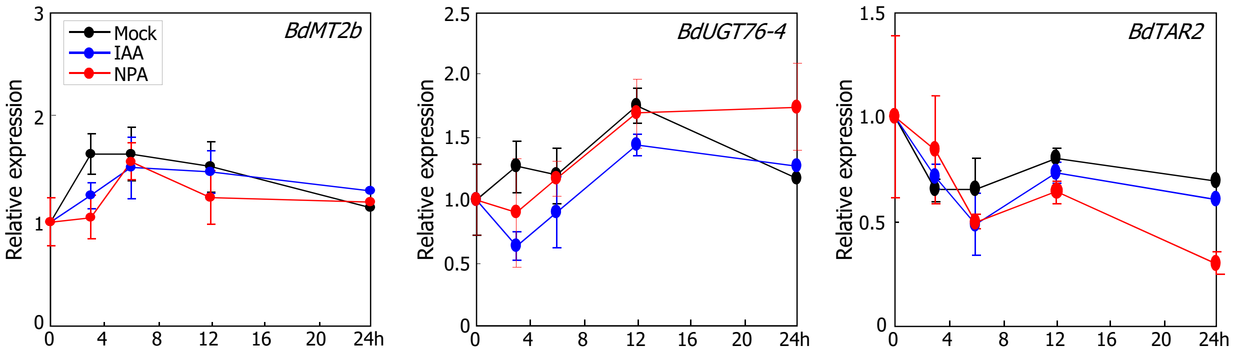

Fig. S13 Effects of auxin and NPA on the transcription of ethylene response genes. Three-week-old plants grown in soil were artificially fallen down to the soil surface, and 0.1 mM IAA or 1 μM NPA solution was sprayed onto the aboveground plant parts. The first leaf nodes and their internodes were harvested at the indicated time points for the extraction of total RNA. Transcript levels were analyzed by RT-qPCR. Biological triplicates, each consisting of fifteen independent plants, were statistically analyzed. Error bars indicate SE.

Supplementary Table 1

Primers			Usage	Sequence
UBC18	(Bradi4g00660)	F	RT-qPCR	5′-GGAGGCACCTCAGGTCATTT
		R	RT-qPCR	5′-ATAGCGGTCATTGTCTTGCG
WOX10	(Bradi3g18800)	F	RT-qPCR	5′-CACGGCATCATGCACTACGG
		R	RT-qPCR	5′-AGGTTTCTGTGTACCGGTGG
WOX11	(Bradi1g18420)	F	RT-qPCR	5′-CTGCTGCTCTCTCGCAATCG
		R	RT-qPCR	5′-AAGACGACCCGGACCCATA
LBD20	(Bradi1g75110)	F	RT-qPCR	5'-AGCTCGCATCCTTCAAGCAG
		R	RT-qPCR	5'-ATTGTTGCCACCGTAAACGC
LBD30	(Bradi1g68170)	F	RT-qPCR	5'-CAGGTGGTGAATCTCCAGGC
		R	RT-qPCR	5'-AAGAGTGCGGAGAGGTCGAT
UGT76-4	(Bradi4g41410)	F	RT-qPCR	5'-ATTCTCCTCTCCGCAACAGA
		R	RT-qPCR	5'-CGGTTAAGCTCCTGCTCTTG
TAR2	(Bradi2g04290)	F	RT-qPCR	5'-GGCTCCATACTACTCTTCGTATC
		R	RT-qPCR	5'-CAGTAGTAGGCCAGGTCGTG
MT2b	(Bradi2g62315)	F	RT-qPCR	5'-CGGAGGAAACTGCAACTGCG
		R	RT-qPCR	5'-GAGGAGGCTGGAGGTCTGGA

Table S1. Primers used. The RT-qPCR primers were designed using the Primer3 software (version 0.4.0, http://primer3.sourceforge.net/releases.php) in a way that they have calculated melting temperatures in a range of 50 - 65 ^{o}C. F and R indicate forward primer and reverse primer, respectively.
