# Supplementary material for: Auxin mediates the touch-induced mechanical stimulation of adventitious root formation under windy conditions in Brachypodium distachyon
Source: BMC Plant Biol. 2020 Jul 16;20:335. doi: 10.1186/s12870-020-02544-8 (PMC7364541; doi:10.1186/s12870-020-02544-8)
Supplement: Supplementary file 2 — Additional file 2 Figure S2. Effects of wind stimulation on the moisture contents of air and soil. [file 12870_2020_2544_MOESM2_ESM.pdf]

Supplementary Figure 2

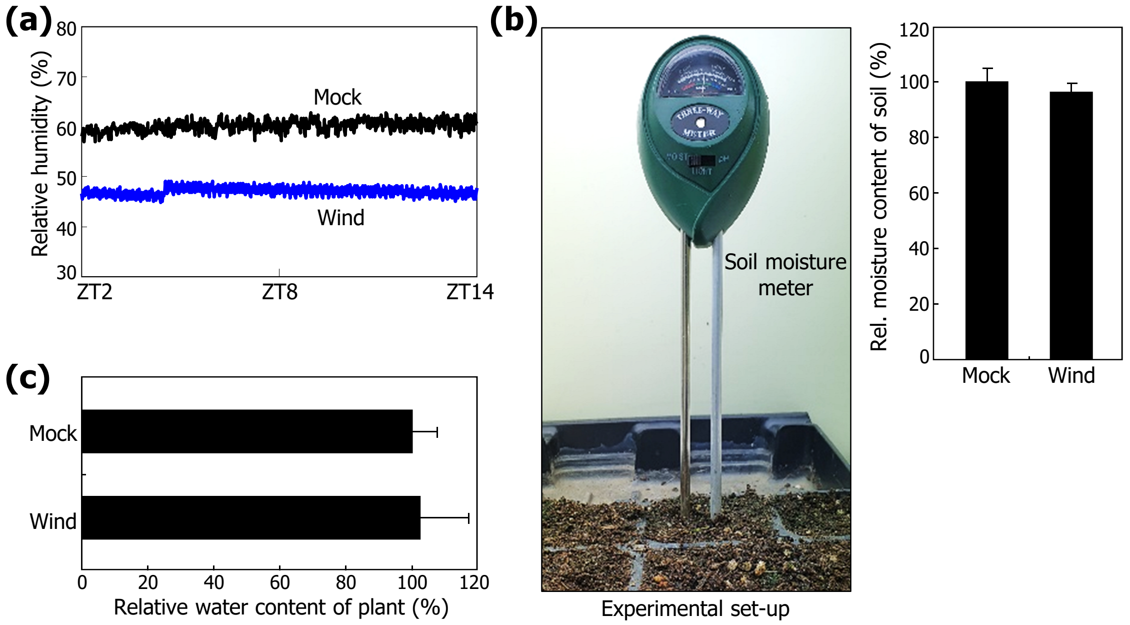

Fig. S2 Effects of wind stimulation on the moisture contents of air and soil. Experimental conditions for the measurements of air humidity and soil moisture are identical to those described in Fig. 2a. **a** Air humidity near the soil surface was recorded during the day. **b** Soil moisture was measured at a depth of 3 cm below the soil surface. Experimental set-up using a soil moisture meter was illustrated (left photograph), and relative soil moistures were measured (right graph). **c** Relative water contents of *Brachypodium* plants during wind treatments were also measured. Three-week-old plants were either grown under mock conditions or exposed to wind stimulation for ten days. Five independent measurements, each consisting of three leaves, were statistically analyzed (*t*-test, $*P < 0.01$). Error bars indicate standard error of the mean (SE).
