# Supplementary material for: Auxin mediates the touch-induced mechanical stimulation of adventitious root formation under windy conditions in Brachypodium distachyon
Source: BMC Plant Biol. 2020 Jul 16;20:335. doi: 10.1186/s12870-020-02544-8 (PMC7364541; doi:10.1186/s12870-020-02544-8)
Supplement: Supplementary file 3 — Additional file 3 Figure S3. Effects of wind flow on air and plant body temperatures. [file 12870_2020_2544_MOESM3_ESM.pdf]

Supplementary Figure 3

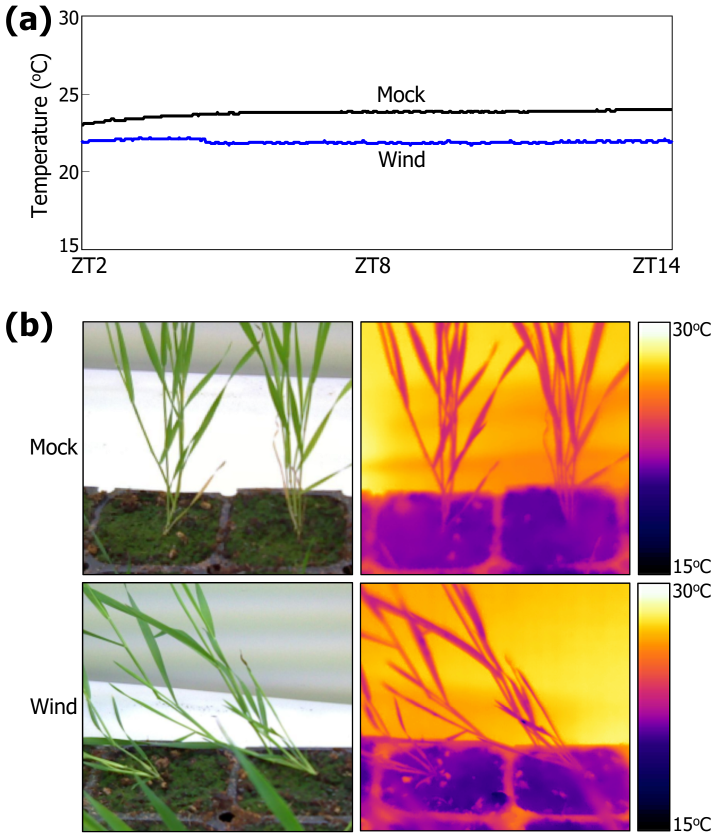

Fig. S3 Effects of wind flow on air and plant body temperatures. Three-week-old plants were either grown under mock conditions (no wind) or exposed to a unidirectional wind flow for varying durations. **a** Air temperatures. Air temperatures near the soil surface were measured during the day. ZT, zeitgeber time. Note that air temperatures under wind treatments are lower by approximately 2 ºC compared to those under mock conditions. **b** Temperature of *Brachypodium* plants. Infrared thermographs of two representative plants were taken at ZT8. Note that plant temperatures were not discernibly altered in the wind-treated plants.
