# Supplementary material for: Auxin mediates the touch-induced mechanical stimulation of adventitious root formation under windy conditions in Brachypodium distachyon
Source: BMC Plant Biol. 2020 Jul 16;20:335. doi: 10.1186/s12870-020-02544-8 (PMC7364541; doi:10.1186/s12870-020-02544-8)
Supplement: Supplementary file 4 — Additional file 4 Figure S4. Physical dissection of wind stimulation. [file 12870_2020_2544_MOESM4_ESM.pdf]

Supplementary Figure 4

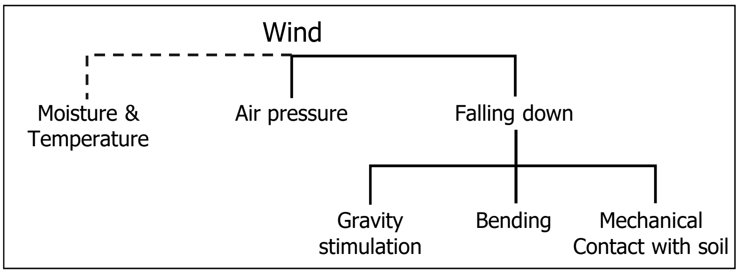

Fig. S4 Physical dissection of wind stimulation. Wind is a complicated mechanical stimulus that can be dissected into various effective factors. The potential effects of individual factors, such as air pressure, gravity stimulation, mechanical bending, and mechanical contact with the soil, on the induction of adventitious root (AR) formation were systematically assayed in this study. Note that air moisture and temperature are frequently affected by wind in nature. However, we found that these two factors are not significantly affected by wind flow in our assay conditions (marked by dashed line).
