# Supplementary material for: Auxin mediates the touch-induced mechanical stimulation of adventitious root formation under windy conditions in Brachypodium distachyon
Source: BMC Plant Biol. 2020 Jul 16;20:335. doi: 10.1186/s12870-020-02544-8 (PMC7364541; doi:10.1186/s12870-020-02544-8)
Supplement: Supplementary file 5 — Additional file 5 Figure S5. Experimental set-up for the lodging phenotypic analysis of Brachypodium plants against mechanical and gravity stimuli. [file 12870_2020_2544_MOESM5_ESM.pdf]

Supplementary Figure 5

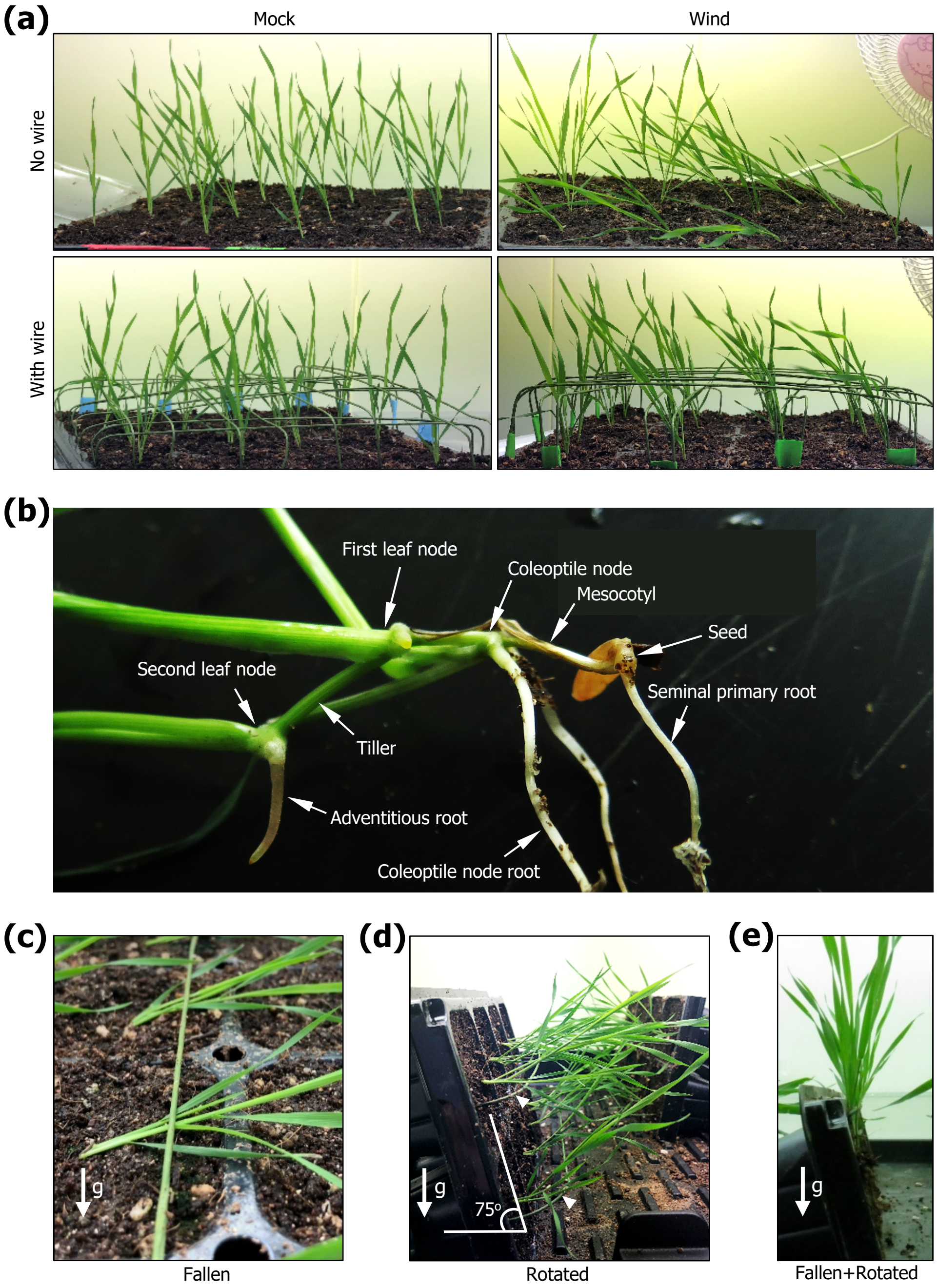

Fig. S5. Experimental set-up for the lodging phenotypic analysis of *Brachypodium* plants against mechanical and gravity stimuli. **a** Experimental set-up for Fig. 3a. **b** Detailed architecture of the *Brachypodium* root system. **c-e** Experimental set-up for 'fallen' and 'rotated' treatments. Plants were artificially fallen down to the soil surface by arresting wires (**c**, Fig. 3b). Plants were also gravi-stimulated by rotating horizontally (**d**, Fig. 3b). To prevent the shoots from falling downward, plants were supported by supporting wires (marked by arrowheads). The supporting wires were arranged carefully not to touch the leaf nodes. Artificially fallen plants were rotated by 75°, allowing them to grow upward (**e**, Fig. 3c). Note that only one side of the leaf nodes was physically contacted with soil particles.
