# Supplementary material for: Auxin mediates the touch-induced mechanical stimulation of adventitious root formation under windy conditions in Brachypodium distachyon
Source: BMC Plant Biol. 2020 Jul 16;20:335. doi: 10.1186/s12870-020-02544-8 (PMC7364541; doi:10.1186/s12870-020-02544-8)
Supplement: Supplementary file 6 — Additional file 6 Figure S6. Effect of falling down without direct soil contact on the induction of AR formation. [file 12870_2020_2544_MOESM6_ESM.pdf]

Supplementary Figure 6

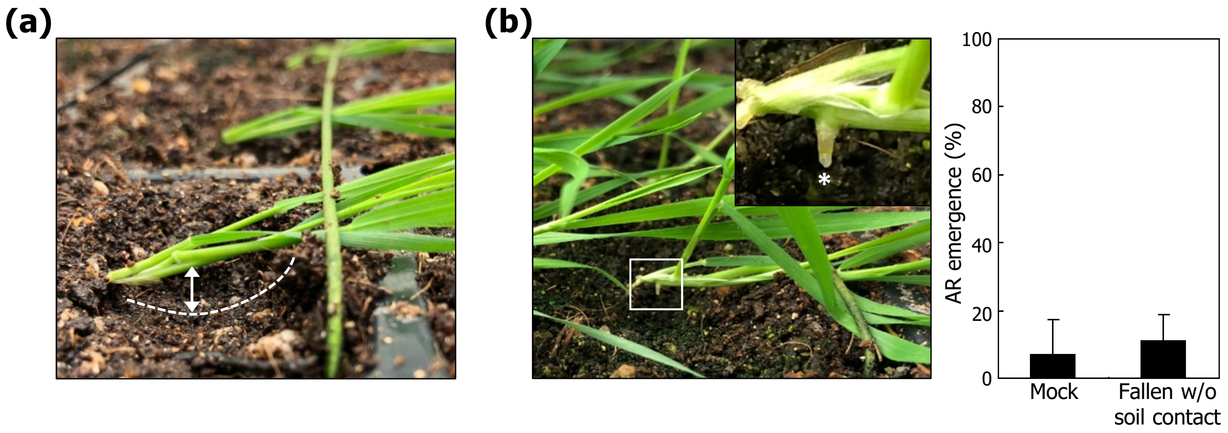

Fig. S6 Effect of falling down without direct soil contact on the induction of AR formation. Three-week-old plants grown in soil were assayed. Three measurements, each consisting of eight plants, were statistically analyzed. Error bars indicate SE. **a** Experimental set-up. Plants were artificially fallen down using arresting wires, and the soil around the leaf nodes were removed so that the leaf nodes does not directly touch the soil particles. **b** Effects of falling down without soil contact on AR emergence. The artificially fallen plants were incubated for ten days, and the number of AR emergence was counted. The inset indicates an enlarged view of the leaf node (marked by a white frame) (left photograph). Asterisk marks an AR primordium. Note that the number of ARs in artificially fallen plants without direct soil contact is similar to that in mock-treated plants (right graph).
