# Supplementary material for: Auxin mediates the touch-induced mechanical stimulation of adventitious root formation under windy conditions in Brachypodium distachyon
Source: BMC Plant Biol. 2020 Jul 16;20:335. doi: 10.1186/s12870-020-02544-8 (PMC7364541; doi:10.1186/s12870-020-02544-8)
Supplement: Supplementary file 7 — Additional file 7 Figure S7. Experimental set-up for the AR phenotypic analysis of plants against mechanical touch. [file 12870_2020_2544_MOESM7_ESM.pdf]

Supplementary Figure 7

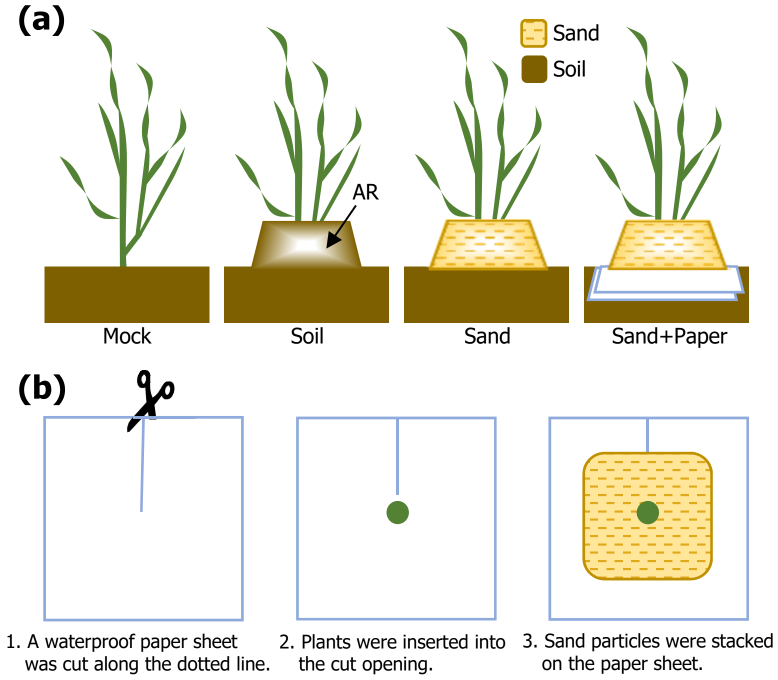

Fig. S7. Experimental set-up for the AR phenotypic analysis of plants against mechanical touch. **a** Experimental set-up for Fig. 4b. The leaf nodes of three-week-old plants grown in soil were covered with either soil or sand layer. In addition, to minimize the moisture of the sand layer, two layers of miracloth were positioned in between the sand and soil layers (sand+paper). It was found that the soil- or sand-covered leaf nodes produce similar numbers of ARs after ten days. **b** Detailed procedure for preparing the sand+paper set-up in **a**.
