# Supplementary material for: Auxin mediates the touch-induced mechanical stimulation of adventitious root formation under windy conditions in Brachypodium distachyon
Source: BMC Plant Biol. 2020 Jul 16;20:335. doi: 10.1186/s12870-020-02544-8 (PMC7364541; doi:10.1186/s12870-020-02544-8)
Supplement: Supplementary file 8 — Additional file 8 Figure S8. Schematic diagram for measuring the moisture content of soil and sand layers. [file 12870_2020_2544_MOESM8_ESM.pdf]

Supplementary Figure 8

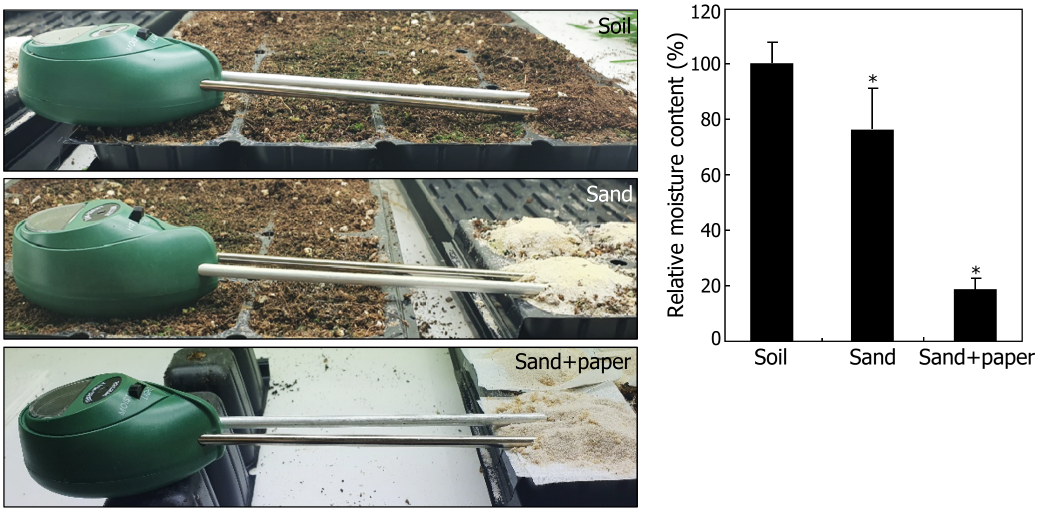

Fig. S8 Schematic diagram for measuring the moisture contents of soil and sand layers. Moisture contents were measured using a soil moisture meter under conditions identical to those described in Fig. 4b. Photographs of experimental set-up described in Fig. 4b were displayed. Three measurements, each consisting of five spots, were statistically analyzed (*t*-test, *$P < 0.01$). Error bars indicate SE.
