# Supplementary material for: Auxin mediates the touch-induced mechanical stimulation of adventitious root formation under windy conditions in Brachypodium distachyon
Source: BMC Plant Biol. 2020 Jul 16;20:335. doi: 10.1186/s12870-020-02544-8 (PMC7364541; doi:10.1186/s12870-020-02544-8)
Supplement: Supplementary file 9 — Additional file 9 Figure S9. Fluorescent imaging of AR primordia in the DII-VENUS reporter plants. [file 12870_2020_2544_MOESM9_ESM.pdf]

Supplementary Figure 9

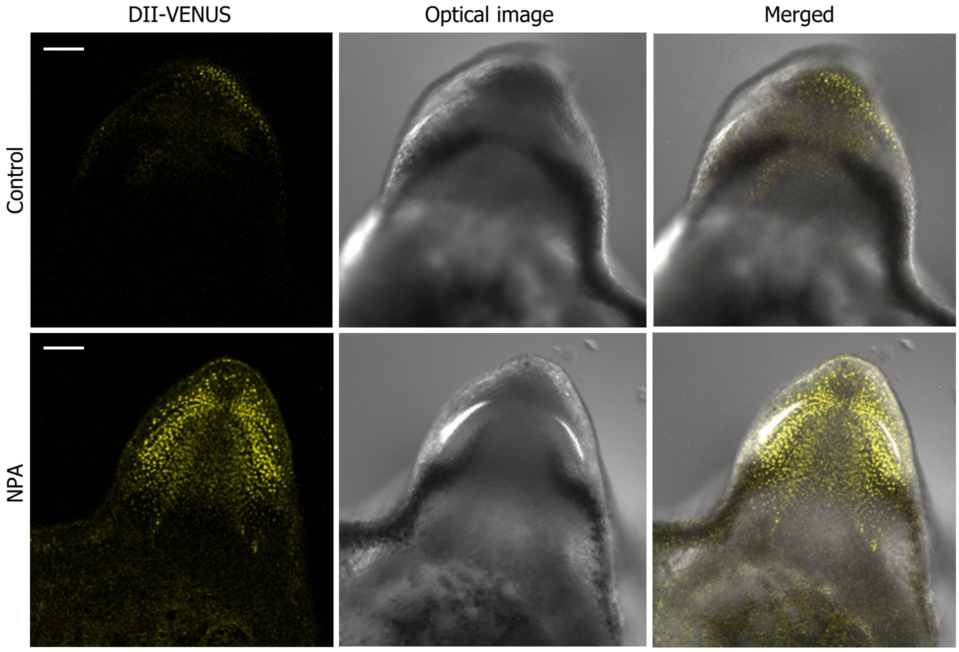

Fig. S9 Fluorescent imaging of AR primordia in the DII-VENUS reporter plants. The *Brachypodium* DII-VENUS reporter plants (*proZmUbi:DII-VENUS*) were employed to verify the negative effects of NPA on auxin accumulation in AR primordia under mechanical stress. The reporter plants have been successfully employed for the analysis of auxin accumulation in *Brachypodium*. Three-week-old reporter plants grown in soil were artificially fallen down, and a NPA solution (1 μM) was sprayed once a day for ten days. Fluorescent images of AR primordia that appeared from the leaf nodes were obtained. In this system, a lower fluorescent intensity represents a higher auxin accumulation, and vice versa. Scale bars, 100 μm.
