# Supplementary material for: Auxin mediates the touch-induced mechanical stimulation of adventitious root formation under windy conditions in Brachypodium distachyon
Source: BMC Plant Biol. 2020 Jul 16;20:335. doi: 10.1186/s12870-020-02544-8 (PMC7364541; doi:10.1186/s12870-020-02544-8)
Supplement: Supplementary file 10 — Additional file 10 Figure S10. Effects of IAA on AR formation. [file 12870_2020_2544_MOESM10_ESM.pdf]

Supplementary Figure 10

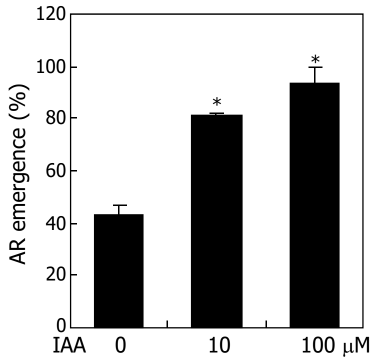

Fig. S10 Effects of IAA on AR formation. Three-week-old plants grown in soil were artificially fallen down, as described in Figure 5, and IAA solutions (either 10 or 100 μM) were sprayed once a day for ten days. Three measurements of AR emergence, each consisting of sixteen plants, were statistically analyzed (*t*-test, $^*P < 0.01$). Error bars indicate SE.
