# Supplementary material for: Auxin mediates the touch-induced mechanical stimulation of adventitious root formation under windy conditions in Brachypodium distachyon
Source: BMC Plant Biol. 2020 Jul 16;20:335. doi: 10.1186/s12870-020-02544-8 (PMC7364541; doi:10.1186/s12870-020-02544-8)
Supplement: Supplementary file 11 — Additional file 11 Figure S11. Effects of ethylene perception inhibitor on the induction of AR formation. [file 12870_2020_2544_MOESM11_ESM.pdf]

Supplementary Figure 11

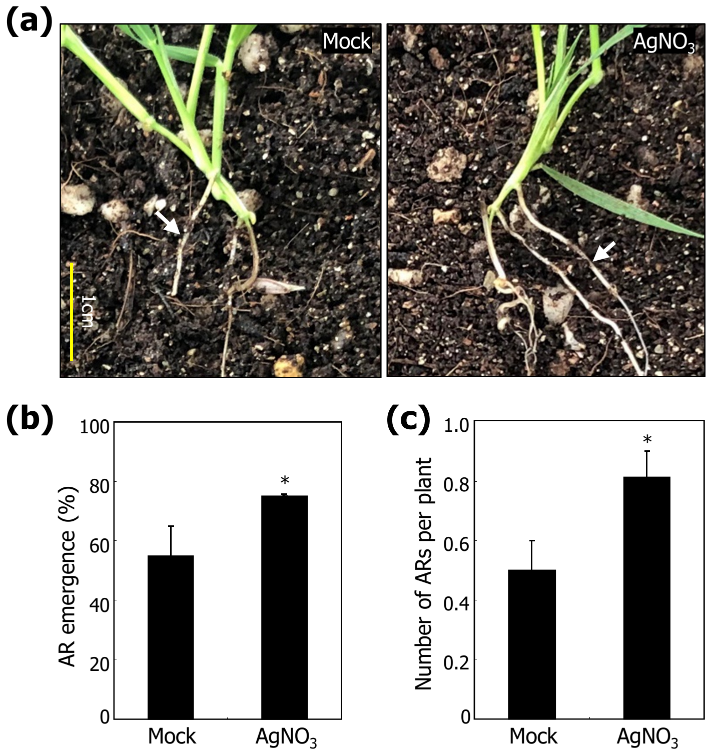

Fig. S11 Effects of ethylene perception inhibitor on the induction of AR formation. Three-week-old plants grown in soil were artificially fallen down, and a solution of 1 mM $AgNO_3$ was sprayed once a day for ten days. **a** Representative plants were photographed. White arrows indicate ARs. **b** AR emergence. **c** Number of ARs per plant. Three experiments, each consisting of sixteen plants, statistically analyzed (*t*-test, *P < 0.01). Error bars indicate SE. Note the slightly promotive effects of $AgNO_3$ on the induction of AR formation, suggesting that ethylene is not a primary determinant of wind-mediated mechano-stimulation on the induction of AR formation.
