# Supplementary material for: Auxin mediates the touch-induced mechanical stimulation of adventitious root formation under windy conditions in Brachypodium distachyon
Source: BMC Plant Biol. 2020 Jul 16;20:335. doi: 10.1186/s12870-020-02544-8 (PMC7364541; doi:10.1186/s12870-020-02544-8)
Supplement: Supplementary file 12 — Additional file 12 Figure S12. Induction of BdWOX and BdLBD genes by soil touch. [file 12870_2020_2544_MOESM12_ESM.pdf]

Supplementary Figure 12

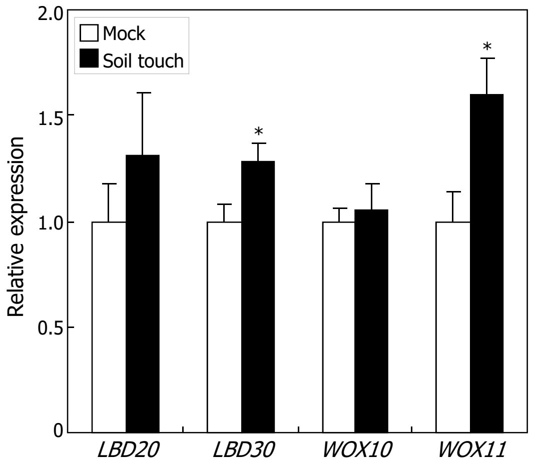

Fig. S12 Induction of *BdWOX* and *BdLBD* genes by soil touch. Following the mechanical stimulation by soil touch, the first leaf nodes and their internodes were harvested for total RNA extraction, and transcript levels were examined by reverse transcription-mediated quantitative PCR (RT-qPCR), as described in Fig. 6b. Biological triplicates, each consisting of eight independent plants, were statistically analyzed (*t*-test, $*P < 0.01$). Error bars indicate SE.
