# Supplementary material for: Auxin mediates the touch-induced mechanical stimulation of adventitious root formation under windy conditions in Brachypodium distachyon
Source: BMC Plant Biol. 2020 Jul 16;20:335. doi: 10.1186/s12870-020-02544-8 (PMC7364541; doi:10.1186/s12870-020-02544-8)
Supplement: Supplementary file 13 — Additional file 13 Figure S13. Effects of auxin and NPA on the transcription of ethylene response genes. [file 12870_2020_2544_MOESM13_ESM.pdf]

Supplementary Figure 13

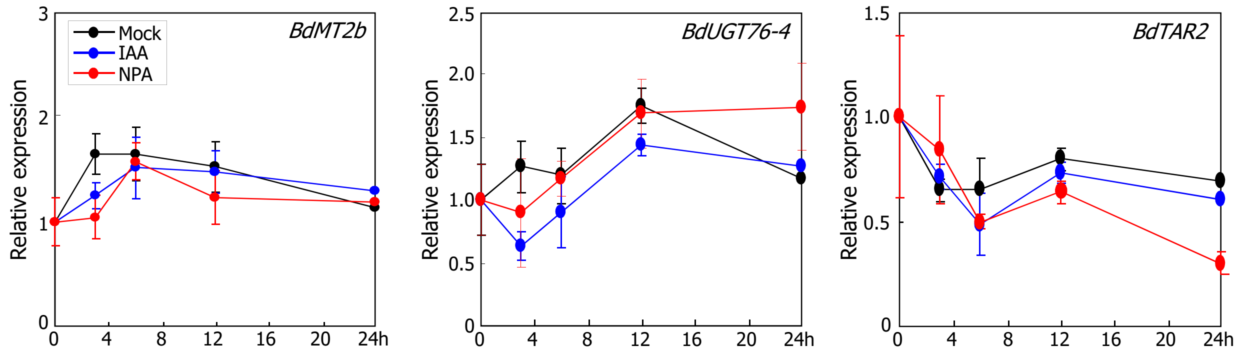

Fig. S13 Effects of auxin and NPA on the transcription of ethylene response genes. Three-week-old plants grown in soil were artificially fallen down to the soil surface, and 0.1 mM IAA or 1 μM NPA solution was sprayed onto the aboveground plant parts. The first leaf nodes and their internodes were harvested at the indicated time points for the extraction of total RNA. Transcript levels were analyzed by RT-qPCR. Biological triplicates, each consisting of fifteen independent plants, were statistically analyzed. Error bars indicate SE.
