# Supplementary material for: Auxin mediates the touch-induced mechanical stimulation of adventitious root formation under windy conditions in Brachypodium distachyon
Source: BMC Plant Biol. 2020 Jul 16;20:335. doi: 10.1186/s12870-020-02544-8 (PMC7364541; doi:10.1186/s12870-020-02544-8)
Supplement: Supplementary file 14 — Additional file 14 Table S1. Primers used. [file 12870_2020_2544_MOESM14_ESM.pdf]

Supplementary Table 1

Primers			Usage	Sequence
UBC18	(Bradi4g00660)	F	RT-qPCR	5′-GGAGGCACCTCAGGTCATTT
		R	RT-qPCR	5′-ATAGCGGTCATTGTCTTGCG
WOX10	(Bradi3g18800)	F	RT-qPCR	5′-CACGGCATCATGCACTACGG
		R	RT-qPCR	5′-AGGTTTCTGTGTACCGGTGG
WOX11	(Bradi1g18420)	F	RT-qPCR	5′-CTGCTGCTCTCTCGCAATCG
		R	RT-qPCR	5′-AAGACGACCCGGACCCATA
LBD20	(Bradi1g75110)	F	RT-qPCR	5'-AGCTCGCATCCTTCAAGCAG
		R	RT-qPCR	5'-ATTGTTGCCACCGTAAACGC
LBD30	(Bradi1g68170)	F	RT-qPCR	5'-CAGGTGGTGAATCTCCAGGC
		R	RT-qPCR	5'-AAGAGTGCGGAGAGGTCGAT
UGT76-4	(Bradi4g41410)	F	RT-qPCR	5'-ATTCTCCTCTCCGCAACAGA
		R	RT-qPCR	5'-CGGTTAAGCTCCTGCTCTTG
TAR2	(Bradi2g04290)	F	RT-qPCR	5'-GGCTCCATACTACTCTTCGTATC
		R	RT-qPCR	5'-CAGTAGTAGGCCAGGTCGTG
MT2b	(Bradi2g62315)	F	RT-qPCR	5'-CGGAGGAAACTGCAACTGCG
		R	RT-qPCR	5'-GAGGAGGCTGGAGGTCTGGA

Table S1. Primers used. The RT-qPCR primers were designed using the Primer3 software (version 0.4.0, http://primer3.sourceforge.net/releases.php) in a way that they have calculated melting temperatures in a range of 50 - 65 ^{o}C. F and R indicate forward primer and reverse primer, respectively.
